# INPP5A phosphatase is a synthetic lethal target in GNAQ and GNA11-mutant melanomas

**DOI:** 10.1038/s43018-023-00710-z

**Published:** 2024-01-17

**Authors:** Ahmed M. O. Elbatsh, Ali Amin-Mansour, Anne Haberkorn, Claudia Textor, Nicolas Ebel, Emilie Renard, Lisa M. Koch, Femke C. Groenveld, Michelle Piquet, Ulrike Naumann, David A. Ruddy, Vincent Romanet, Julia M. Martínez Gómez, Matthew D. Shirley, Peter Wipfli, Christian Schnell, Markus Wartmann, Martin Rausch, Martine J. Jager, Mitchell P. Levesque, Sauveur-Michel Maira, Eusebio Manchado

**Affiliations:** 1 Oncology, Novartis Institute for Biomedical Research, Basel, Switzerland; 2 Oncology, Novartis Institute for Biomedical Research, Cambridge, MA USA; 3 PK Sciences, Novartis Institute for Biomedical Research, Basel, Switzerland; 4 Chemical Biology and Therapeutics, Novartis Institute for Biomedical Research, Basel, Switzerland; 5 Dermatology Department, University Hospital Zurich, University of Zurich, Zurich, Switzerland; 6 Department of Ophthalmology, Leiden University Medical Center, Leiden, The Netherlands

**Keywords:** Cancer genetics, Cancer, Eye cancer

## Abstract

Activating mutations in *GNAQ/GNA11* occur in over 90% of uveal melanomas (UMs), the most lethal melanoma subtype; however, targeting these oncogenes has proven challenging and inhibiting their downstream effectors show limited clinical efficacy. Here, we performed genome-scale CRISPR screens along with computational analyses of cancer dependency and gene expression datasets to identify the inositol-metabolizing phosphatase INPP5A as a selective dependency in GNAQ/11-mutant UM cells in vitro and in vivo. Mutant cells intrinsically produce high levels of the second messenger inositol 1,4,5 trisphosphate (IP3) that accumulate upon suppression of INPP5A, resulting in hyperactivation of IP3-receptor signaling, increased cytosolic calcium and p53-dependent apoptosis. Finally, we show that GNAQ/11-mutant UM cells and patients’ tumors exhibit elevated levels of IP4, a biomarker of enhanced IP3 production; these high levels are abolished by GNAQ/11 inhibition and correlate with sensitivity to INPP5A depletion. Our findings uncover INPP5A as a synthetic lethal vulnerability and a potential therapeutic target for GNAQ/11-mutant-driven cancers.

## Abstract (web summary)

Manchado and colleagues combine CRISPR screening and transcriptomics to identify INPP5A as a dependency and therapeutic target in uveal melanoma driven by mutations in GNAQ/GNA11 and show that IP4 levels correlate with sensitivity to INPP5A loss.

## Main

UM is the most recurring primary intraocular cancer in adults and the second most common melanoma subtype after cutaneous skin melanoma (CM)^1^. Despite effective clinical management of the primary disease, 50% of patients with UM develop distant metastases and have an overall survival of less than 12 months^2^. Localized primary tumors can be controlled by radiotherapy, phototherapy or enucleation, whereas metastatic malignancies are refractory to chemotherapy, MAPK-targeted inhibitors or immune-checkpoint blockades^2^. Although both arise from melanocytes, uveal and cutaneous melanomas are highly distinct genetically and clinically^3^. UM tumors do not harbor mutations in *BRAF* or *NRAS*, the two most prevalent oncogenes mutations in CM^4^. Instead, mutually exclusive activating mutations in *GNAQ* and *GNA11*, encoding GNAQ and GNA11 G-proteins, occur in about 90% of UMs^5,6^. The remaining 10% are driven by mutations identified in the GNAQ/11-linked G-protein-coupled receptor (GPCR), cysteinyl leukotriene receptor 2 (*CYSLTR2*) or phospholipase C β4 (*PLCβ4*), a direct downstream effector of GNAQ/11 (refs. ^7,8^).

GNAQ/11 are the α subunits of the heterotrimeric Gq-protein family^9^. These highly homologous proteins function as the core GTPase machinery that relays signals from upstream GPCRs to downstream effectors to modulate numerous cellular processes^10^. Oncogenic mutations of GNAQ/11, occurring at residues Q209 (95%) and R183 (5%), give rise to GTPase-defective proteins that lack their intrinsic GTP-hydrolyzing activity^1^. These oncoproteins are thus predominantly locked in an active configuration, resulting in constitutive activation of their downstream signaling^11^. GNAQ/11 downstream effector, PLCβ, hydrolyzes phosphatidylinositol 4,5-bisphosphate (PIP2) at the plasma membrane to produce two cellularly active second messengers; IP3 and diacylglycerol (DAG)^12^. IP3 mobilizes calcium to regulate various intracellular processes, whereas DAG is responsible for the plasma membrane tethering of protein kinase C (PKC), a serine/threonine kinase that is required for MAPK pathway activation^13^. In principle, all known UM oncogenic mutations lead to the constitutive production of both second messengers. While it is conceivable that DAG is essential for UM cells to activate the MAPK pathway to promote cell proliferation, the cellular and molecular implications of the sustained production of IP3 in the context of UM are largely unknown.

Unprecedented advances in high-throughput functional genomics and sequencing technologies have permitted the execution of large-scale profiling of almost all cancer types. Such projects generate extensive datasets that represent a rich repository to discover cancer-specific genetic vulnerabilities and unravel mechanisms of oncogenesis. In this study, we performed genome-scale CRISPR knockout screens on UM cells and analyzed publicly available cancer dependency and expression datasets to identify GNAQ/11-mutant-specific dependencies. We found inositol polyphosphate 5-phosphatase A (INPP5A) to be selectively required for the growth of GNAQ/11-mutant cells (GNAQ/11^Mut^) in vitro and in vivo, yet dispensable for GNAQ/11 wild-type (GNAQ/11^WT^) cells. In addition, we uncovered the underlying molecular mechanism of INPP5A dependency in GNAQ/11-mutant cells. Finally, we identified inositol tetrakisphosphate (IP4) as a predictive biomarker for INPP5A dependency and showed that it is elevated in GNAQ/11^Mut^ patient-derived xenografts and human tumors.

## Results

### Genetic screening for GNAQ/11-mutant UM dependencies

**Fig. 1:**
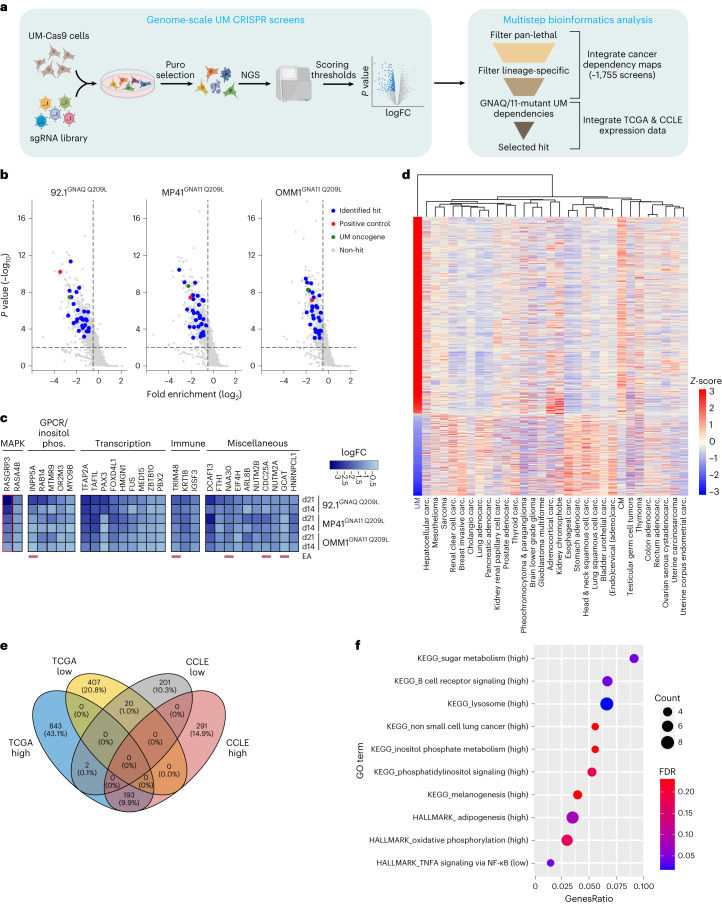
Genome-scale CRISPR-Cas9 screens identify genetic dependencies in GNAQ/11-mutant UM cells. **a**, Schematic of the integrative approach used to identify specific genetic dependencies in GNAQ/11-mutant UM cells. FC, fold change; NGS, next-generation sequencing. **b**, Gene-level fold enrichment of sgRNAs in UM cell lines (*x* axis) and one-sided *P* values at day 21 (*y* axis). Dashed lines indicate significance (*P* < 0.01) and fold enrichment ≤−0.5. Positive control (*RASGRP3*) is depicted in red and driver oncogenes (*GNAQ* or *GNAQ11*) are in green. Genes that passed all filtering steps (Extended Data Fig. 1c) on both days 14 and 21 (*n* = 29 genes) are shown in blue. **c**, Heat map depicting fold enrichment of identified genes in **b**. The positive control (*RASGRP3*) is highlighted in red. Genes are categorized based on biological function. Genes encoding proteins with enzymatic activity are underlined. Inositol phos., inositol phosphate biology; EA, enzymatic activity. **d**, Heat map depicting median per tumor group mRNA expression *z*-scores (*y* axis) in the indicated solid cancers (*x* axis) analyzed from TCGA dataset. Dendrogram shows the clustering of tumor types based on the gene expression profiles. Carc., carcinoma. **e**, Venn diagram showing the overlap of differentially expressed genes (high, highly expressed genes; low, lowly expressed genes) in UM compared to other solid cancers analyzed from TCGA and CCLE datasets. **f**, Dot plot displaying MSigDB KEGG and HALLMARK pathways enrichment analysis for highly expressed (high) and lowly expressed (low) genes in UM. Dot color depicts the false discovery rate (FDR) value and dot size indicates the number of genes overlapping with the pathway gene list. Source data

**Extended Data Fig. 1:**
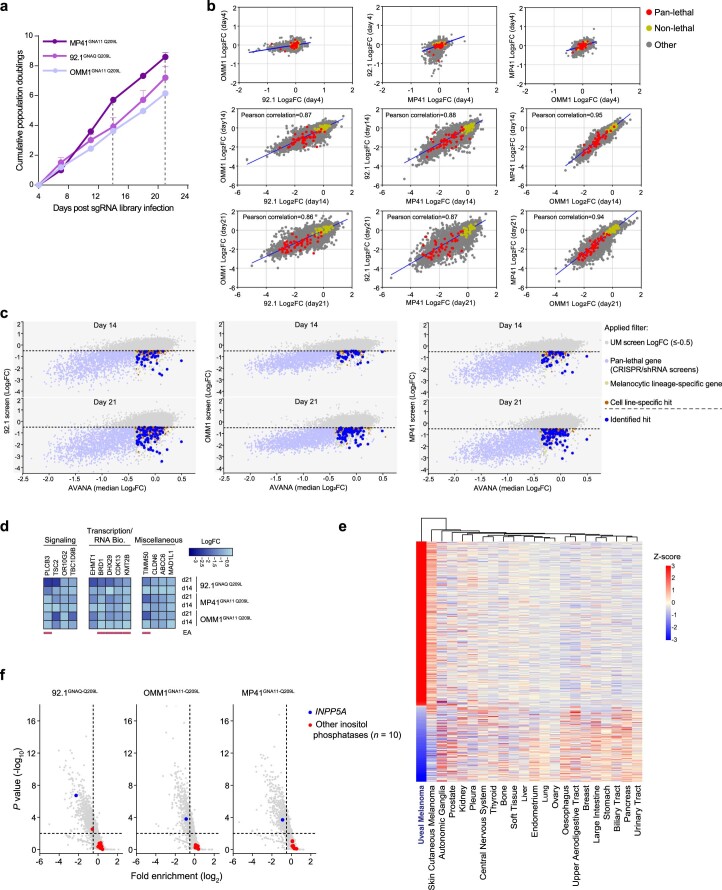
Genome-scale CRISPR KO screens in 92.1, MP41 and OMM1 UM cell lines. **a**, Graph depicting number of cell doublings of the indicated cell lines over the screens’ durations. Cells number counting started on day 4 after seeding for the expansion phase of the screen following the completion of puromycin selection. Cells were harvested and counted every 3 to 4 days. Dashed lines indicate harvesting time points for genomic DNA isolation on days 4, 14 and 21. Data presented as mean ± s.e.m.; *n* = 3 biologically independent samples. **b**, Scatter plots of log2-transformed gene-level fold enrichment of the indicated cell lines on days 4, 14 and 21 of the CRISPR-Cas9 screens. Correlation between the 3 screens on days 14 and 21 is shown. The high degree of correlation between the screens performed on different cell lines highlights the reproducibility of the screens. Red, light green and gray dots depict pan-lethal, nonlethal, and all other genes, respectively. Approximately 50 pan-lethal and 50 nonlethal genes were unbiasedly selected as described^67^. Nonlethal and pan-lethal genes overlapped on day 4. On days 14 and 21, pan-lethal genes were strongly depleted from the bulk population while nonlethal genes remained largely unchanged compared to day 4 indicating the technical success of the screens. **c**, Scatter plots of median log2-transformed gene-level fold enrichment of CRISPR-Cas9 screens performed with project AVANA (*x* axis) and 92.1 screen (left), OMM1 screen (middle) and MP41 screen (right) performed in this study at days 14 (top panel) and 21 (bottom panel) (*y* axis). Different dot colors depict genes that were removed at each filtering step of the analysis pipeline used to select the final gene list. Dashed lines represent the scoring threshold of the Log2FC of UM screens (≤ −0.5). Blue dots represent final hits. **d**, Heat map depicting fold enrichment of genes that passed all filters shown in (**c**) only on day 21 but not on day 14 (*n* = 13). Genes are categorized based on their biological function. Genes encoding proteins with enzymatic activity are underlined. EA, enzymatic activity. **e**, Heat map depicting median per tumor group mRNA expression z-scores (*y* axis) in UM cell lines compared to all other cell lines of solid tumors analyzed from CCLE expression dataset (*x* axis). Cancer types represented by less than 5 cell lines were excluded to account for statistical power. Dendrogram shows the clustering of cell lines based on the expression profiles of all genes. **f**, Gene-level fold enrichment of sgRNAs of UM CRISPR-Cas9 screens (*x* axis) and one-sided *P* values at day 21 (*y* axis). Dashed lines indicate significance (*P* < 0.01) and LogFC (≤ −0.5). Blue dots represent *INPP5A* and red dots represent other inositol phosphatases encoded in the human genome (*INPP1*, *INPP4A*, *INPP4B*, *INPP5B*, *INPP5D*, *INPP5E*, *INPP5F*, *INPP5J*, *INPP5K* and *INPPL1*). Source data

To probe selective genetic dependencies in UM cells with *GNAQ/11* mutations, we performed genome-scale CRISPR-Cas9 screens in three independent GNAQ/11^Mut^ UM cell lines and concurrently analyzed datasets of projects AVANA and Score (performed using CRISPR-Cas9 technology) and projects Achilles and DRIVE (performed using RNA interference (shRNA) technology, respectively)^14–17^. In addition, we interrogated the gene expression data generated by Cancer Cell Line Encyclopedia (CCLE) and The Cancer Genome Atlas (TCGA) (Fig. 1a)^18,19^. Cas9-expressing 92.1, OMM1 and MP41 UM cell lines were transduced with a single-guide RNA (sgRNA) library and changes in library representation after 4, 14 and 21 d of culture were monitored using deep sequencing (Extended Data Fig. 1a). sgRNA read count distribution and depletion patterns of sgRNAs targeting known pan-lethal and nonlethal genes were comparable among the three screens, reflecting the overall high quality of our screening approach (Extended Data Fig. 1b). *GNAQ* and *GNA11* sgRNAs were only depleted in cell lines harboring an oncogenic mutation in the corresponding gene, confirming that UM cells depend on continuous GNAQ or GNA11 activity (Fig. 1b).

We then integrated the data of the three UM screens with publicly available cancer dependency maps using a multilayer bioinformatics analysis pipeline. The analysis entailed stepwise filtering criteria that ultimately led to the identification of essential genes specifically for GNAQ/11^Mut^ cells while excluding pan-cancer and melanocyte-lineage lethal genes (Fig. 1a and Extended Data Fig. 1c). In total, we identified 29 genes that passed all filtering steps at two time points (day 14 and day 21) and an additional 13 genes that scored only on day 21 (Fig. 1c and Extended Data Fig. 1d). The robustness of our approach was supported by the identification of *RASGRP3*, a validated GNAQ/11^Mut^ UM genetic dependency (Fig. 1b,c)^20^. Functional annotation of the identified hits revealed their involvement in diverse biological processes, including MAPK and GPCR signaling, transcription regulation, immune response and inositol phosphate metabolism (Fig. 1c, Extended Data Fig. 1d and Supplementary Table 1).

To prioritize our gene list, we mapped the expression profile of GNAQ/11^Mut^ UM by leveraging the messenger RNA gene expression datasets of CCLE and TCGA. We defined a gene expression signature for GNAQ/11^Mut^ UM consisting of 213 genes (Supplementary Table 2). Of these, 193 and 20 genes displayed a significantly higher and lower mRNA expression in UM compared to all other solid tumor types, respectively (Fig. 1d,e and Extended Data Fig. 1e). Consistent with previous reports, Gene Ontology (GO) term enrichment analysis revealed processes that included melanogenesis, inflammatory response and oxidative phosphorylation, as well as enrichment of genes involved in inositol phosphate metabolism (Fig. 1f)^21,22^. Notably, a few genes in the hit list of the genetic screens belong to this metabolic pathway, highlighting the relevance of inositol phosphate biology in GNAQ/11^Mut^ UM cells (Fig. 1c,f). Among these, inositol polyphosphate 5-phosphatase A (*INPP5A*) emerged as one of the top candidate genes (Fig. 1c). We thereby decided to follow up on *INPP5A* for several reasons. First, our comprehensive analysis revealed its selective essentiality in GNAQ/11^Mut^ UM, whereas none of the ten other inositol phosphatase genes encoded in the human genome was preferentially essential (Extended Data Fig. 1f). Second, the role of *INPP5A* in UM remains largely unknown. Last, INPP5A de facto is an enzyme, hence may represent an appealing druggable vulnerability in UM.

### INPP5A is required for the growth of GNAQ/11-mutant UM

**Fig. 2:**
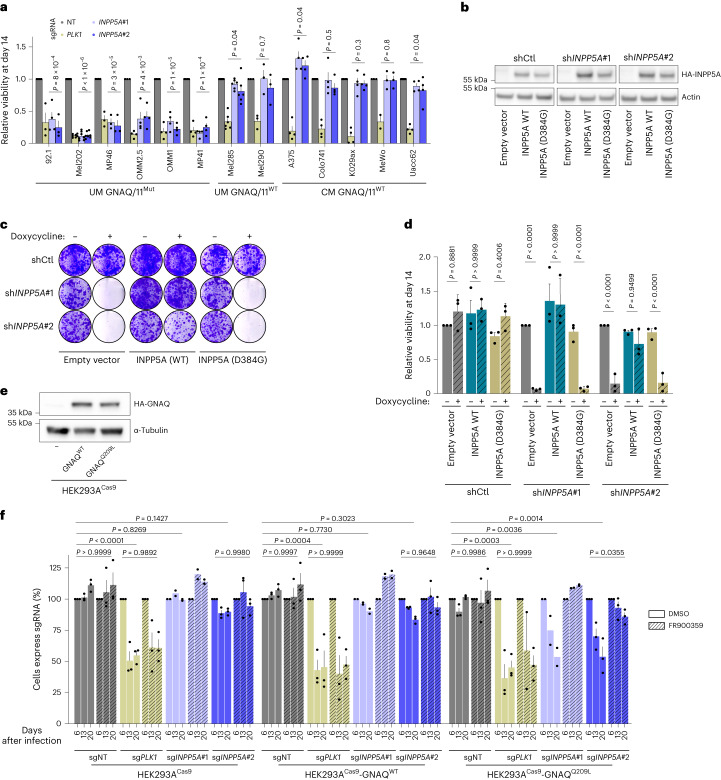
INPP5A is synthetic lethal with GNAQ/11 oncogenic mutations. **a**, Relative viability of the depicted cell lines 14 d following sgRNA transduction. Two sgRNAs against *INPP5A*, sg*INPP5A*#1 and sg*INPP5A*#2; negative control, non-targeting (NT); and positive control, *PLK1* sgRNAs were used (all cell lines *n* = 4, MP41, MeWo and Mel290 *n* = 3, Mel285 *n* = 6, and Mel202 *n* = 7 biologically independent samples were used). **b**, Immunoblot showing expression levels of empty expression vector, HA-INPP5A wild-type or HA-INPP5A-D384G (phosphatase-deficient) in 92.1 UM cells transduced with the indicated sgRNAs. **c**, Clonogenic growth of cell lines shown in **b** 14 d following the induction of shRNA expression by doxycycline (*n* = 3 independent experiments). shCtl, control shRNA. **d**, Relative viability of the depicted cell lines 14 d following the induction of shRNA expression by doxycycline, *n* = 3 independent experiments. **e**, Immunoblot showing expression levels of HA-GNAQ in HEK293A-Cas9 parental cells or cells expressing HA-GNAQ^WT^ or HA-GNAQ^Q209L^ cDNAs. **f**, Competition-based proliferation assay in HEK293A-Cas9 parental cells or cells expressing the indicated HA-GNAQ cDNAs treated with dimethylsulfoxide (DMSO) or GNAQ/11 inhibitor (FR900359 10 nM). Percentage of cells expressing sgRNAs (mCherry^+^ cells) at the indicated time points are shown, *n* = 3 biologically independent samples for all conditions except for sg*INPP5A*#1 *n* = 2. All data are presented as mean ± s.e.m.; *P* values were determined using an unpaired Student’s *t*-test with multiple comparisons (**a**) or two-way analysis of variance (ANOVA) with multiple comparisons (**d**,**f**). Western blots were repeated twice and representative experiments are shown. Source data

**Extended Data Fig. 2:**
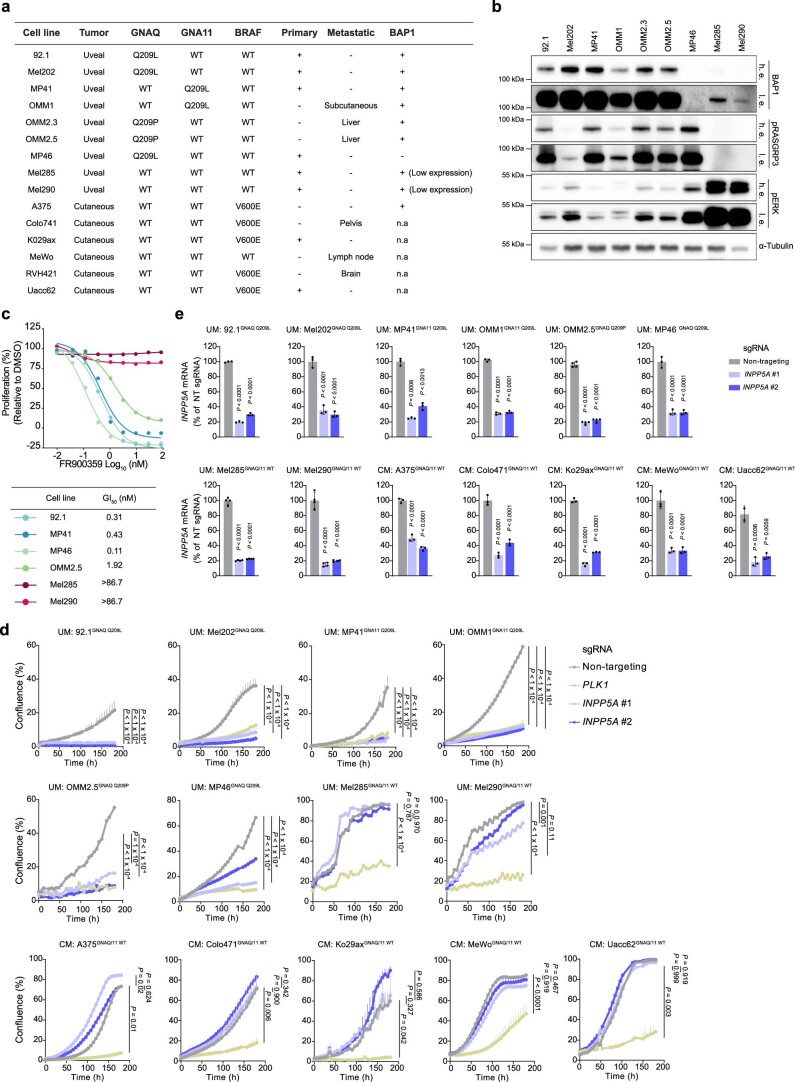
INPP5A is essential for the survival of uveal melanoma cells. **a**, Table summarizing relevant information of all cell lines used to conduct the experiments of this study. OMM2.3 and OMM2.5 cell lines are derived from liver metastatic UM tumors of the same patient. Only OMM2.5 was used in this study. **b**, Immunoblot showing levels of BAP1, pRASGRP3 and pERK in the indicated cell lines. BAP1 is a tumor suppressor that is known to be frequently mutated in metastatic UM tumors and its loss is associated with poor prognosis^68^. l.e., low exposure, h.e., high exposure. **c**, Proliferation percentage of the indicated cell lines relative to DMSO treatment upon the inhibition of GNAQ/11 activity with FR900359 (FR) for 5 days (top). Growth inhibition 50 (GI50, nM) values for each cell line is shown (bottom). Inhibition of GNAQ/11 activity with FR suppressed the proliferation of 92.1 (GNAQ-Q209L), MP41 (GNA11-Q209L), MP46 (GNAQ-Q209L, BAP1 null), and OMM2.5 (GNAQ-Q209P, liver metastatic) cell lines, but did not affect the proliferation of the GNAQ/11^WT^ cell lines, Mel285 or Mel290, *n* = 2 independent experiments. **d**, Growth curves of the depicted cell lines determined by Incucyte live-cell analysis after transduction with indicated sgRNAs. *PLK1* knockout (positive control gene) impacted the proliferation of all cell lines indicating that the CRISPR-Cas9 system is functional in all cell lines, while *INPP5A* depletion only affected the growth of GNAQ/11^Mut^ UM cell lines. *n* = 3 independent experiments for all cell lines, except A375, Mel285, Mel290 and OMM2.5 *n* = 2 and MP46 *n* = 1 with two technical replicates. NT, non-targeting. **e**, Quantitative real-time PCR (qRT–PCR) analysis for *INPP5A* mRNA levels of the depicted cell lines 72 h after transduction with the indicated sgRNAs. mRNA levels are shown as percentage of NT sgRNA. A representative of *n* = 3 biologically independent samples for all cell lines, except OMM2.5, Mel285 and Mel290 *n* = 4 biologically independent samples is shown. NT, non-targeting. All data are presented as mean ± s.e.m.; *P* values were determined by one-way analysis of variance (ANOVA) with multiple comparisons (**d**, **e**). Western blot was repeated four times with similar results. Source data

**Extended Data Fig. 3:**
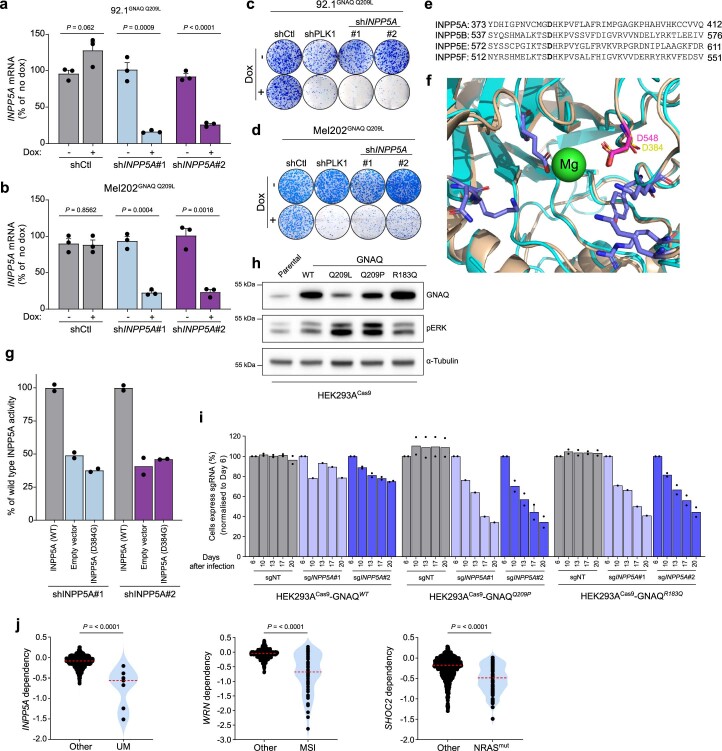
INPP5A is synthetic lethal with different GNAQ oncogenic mutations. **a**,**b**, Quantitative real-time PCR (qRT–PCR) analysis for *INPP5A* mRNA levels of doxycycline-inducible-shRNA 92.1 (**a**) Mel202 (**b**) cells 72 h after doxycycline treatment shown as percentage of no doxycycline control, *n* = 3 biologically independent samples. Dox, doxycycline, shCtl, negative control shRNA. **c**,**d**, Clonogenic growth of doxycycline-inducible shRNA 92.1 (**c**) and Mel202 (**d**) cells 14 days following doxycycline treatment. A representative of *n* = 3 independent experiments is shown. Dox, doxycycline. **e**, Sequence alignment of a specific region of the catalytic phosphatase domain of the indicated human 5-phosphatase enzymes. The conserved aspartate residue (Asp384 in INPP5A) which is predicted to be essential for the catalytic activity of the 5-phosphatases is highlighted in bold^24^. Missense mutations in this residue are found in *INPP5F* (*OCRL*) in Lowe Syndrome and Dent 2 Disease patients and are known to reduce the enzymatic activity of OCRL^69^. **f**, Structures of a conserved region of the catalytic domains of INPP5B (PBD ID: 3MTC) and INPP5A (structural model) superimposed. The model shows the overlap between the two aspartate residues of INPP5A (Asp384) and INPP5B (Asp548) highlighted in (**e**). As indicated in INPP5B structure, Asp548 is directly involved in hydrolysis of the phosphate group and thus is critical for the catalytic activity of this class of phosphatase enzymes. The homologous residue in INPP5A, Asp384, is used as a phosphatase-deficient mutant in this study. **g**, Phosphatase activity assay performed with cell lysates of 92.1-doxycycline-inducible shRNA cells expressing the indicated INPP5A cDNAs 72 h after doxycycline treatment. Inositol 1,4,5 trisphosphate was added as a substrate, and free inorganic phosphate levels were measured and plotted as percentage of wild-type INPP5A, *n* = 2 biologically independent samples. **h**, Immunoblot showing levels of GNAQ, pERK and α-tubulin in HEK293A-Cas9 parental cells or cells expressing the indicated GNAQ cDNAs. GNAQ levels are the total of endogenous and exogenously expressed GNAQ. pERK indicates the signaling output of GNAQ activity. **i**, Competition-based proliferation assay in HEK293A-Cas9 cells expressing wild-type, Q209P or R183Q mutant HA-GNAQ. Cells were transduced with the depicted sgRNAs and the percentage of cells expressing sgRNAs (GFP^+^ or mCherry^+^ cells) at the indicated time points normalized to day 6 post-transduction are shown, *n* = 2 biologically independent samples. **j**, Comparison of *INPP5A* - GNAQ/11^Mut^ UM synthetic lethal interaction to other known synthetic lethal pairs. Dependency score of left: *INPP5A* in UM, *n* = 7 UM and *n* = 932 other non-UM cell lines. Middle: *WRN* in microsatellite instable (MSI) cell lines, *n* = 51 MSI and *n* = 541 MSS microsatellite stable cell lines. Right: *SHOC2* in *NRAS* Q61R mutant cell lines, *n* = 63 mutant cell lines and *n* = 878 wild-type cell lines. Width of colored regions represents density estimates. Dashed red lines represent mean dependency score. Data obtained from DepMap v2022Q1. *INPP5A* dependency data is a combination of data obtained from AVANA CRISPR screens and the 3 UM CRISPR-Cas9 screens performed in this study. All data are presented as mean ± s.e.m.; *P* values were determined by one-way analysis of variance (ANOVA) with multiple comparisons (**a**, **b**) or two-tailed, unpaired Student’s *t*-test (**j**). Western blot was repeated twice with similar results. Source data

To validate *INPP5A* dependency, we evaluated the effect of *INPP5A* knockout in six GNAQ/11^Mut^ UM and seven GNAQ/11^WT^ UM and CM cell lines using two individual *INPP5A* sgRNAs (Extended Data Fig. 2a–c and Supplementary Table 3). Depleting *INPP5A* dramatically inhibited the clonogenic growth as well as the proliferation of all GNAQ/11^Mut^ UM cell lines to a similar effect as compared to the positive control, *PLK1* (Fig. 2a and Extended Data Fig. 2d). In contrast, *INPP5A* knockout did not affect the viability of GNAQ/11^WT^ UM or CM cells, albeit comparable depletion efficiency among all cell lines (Extended Data Fig. 2e). We also transduced two UM cell lines with doxycycline-inducible *INPP5A* shRNAs (Extended Data Fig. 3a,b). Complementing the CRISPR-Cas9 data, knocking down *INPP5A* markedly diminished the viability of the mutant cells (Extended Data Fig. 3c,d). These results indicate that INPP5A is selectively essential for the viability of GNAQ/11^Mut^ UM cells.

Our findings suggest that INPP5A dependence in GNAQ/11^Mut^ cells could likely be exploited through INPP5A inhibition. To assess whether the enzymatic activity of INPP5A is essential for GNAQ/11^Mut^ UM, we expressed shRNA-insensitive complementary DNA of either wild-type or phosphatase-deficient INPP5A in the doxycycline-inducible sh*INPP5A* 92.1 cells (Fig. 2b). To generate this catalytic mutant, we introduced a missense mutation (Asp384Gly) in a highly conserved residue essential for the phosphatase activity of INPP5A (Extended Data Fig. 3e–g)^23,24^. In cDNA complementation experiments, wild-type INPP5A rescued the lethality induced by depleting the endogenous protein, confirming that the lethal phenotype accompanying INPP5A loss is a consequence of its on-target inactivation (Fig. 2c,d). In stark contrast, the catalytic mutant failed to rescue the lethal phenotype, suggesting that the phosphatase activity of INPP5A is essential to maintaining the viability of GNAQ/11^Mut^ UM and can be exploited as a potential therapeutic target.

To further investigate the functional relationship between INPP5A and GNAQ/11 oncoproteins in an UM lineage-independent context, we expressed GNAQ^WT^ or different mutants of GNAQ (Q209L, Q209P or R183Q) in HEK293A-Cas9 cells and assessed the effect of *INPP5A* knockout using competition-based proliferation assays (Fig. 2e and Extended Data Fig. 3h). INPP5A depletion inhibited the proliferation of all GNAQ^Mut^-expressing cells but imposed negligible effects on the parental or GNAQ^WT^ HEK293A cells (Fig. 2f and Extended Data Fig. 3i). Suppressing GNAQ signaling activity using the GNAQ/11-specific inhibitor FR900359 (FR) rescued the lethality evoked by INPP5A depletion in the mutant cells (Fig. 2f)^11^. Taken together, our findings reveal a lineage-independent synthetic lethal relationship between INPP5A loss and GNAQ/11 oncogenic mutants mediated through the aberrant signaling of these oncoproteins. Notably, INPP5A and mutant GNAQ/11 interaction compared favorably to other known synthetic lethal pairs such as NRAS mutants and SHOC2 or WRN helicase in microsatellite-instable cells (Extended Data Fig. 3j)^25,26^.

### IP3 signaling drives INPP5A dependency in GNAQ/11-mutant UM

**Fig. 3:**
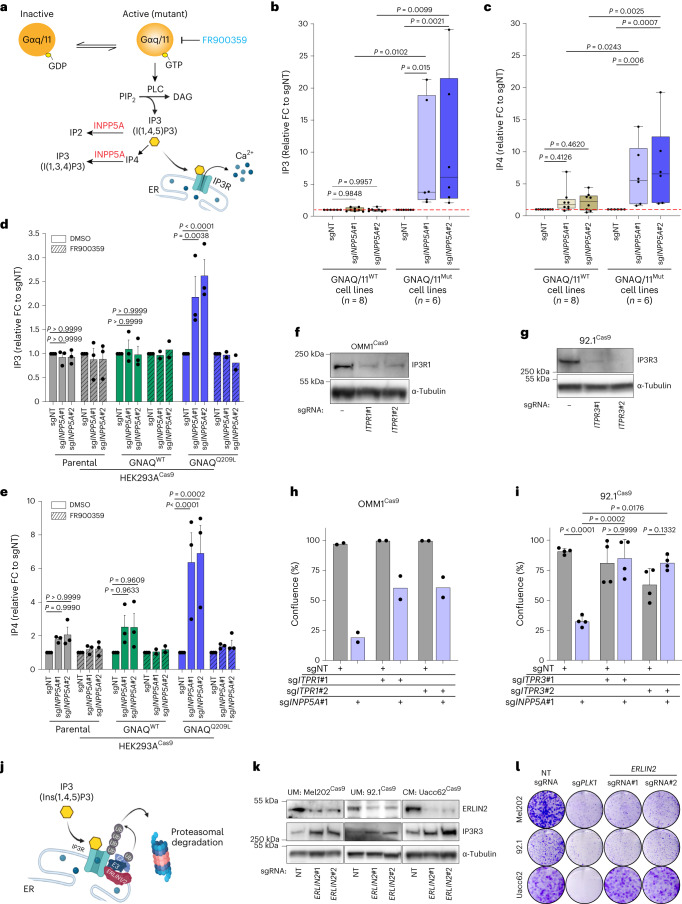
Enhanced IP3 signaling drives INPP5A dependency in GNAQ/11-mutant UM cells. **a**, Schematic depiction of INPP5A-mediated metabolic flux of inositol phosphates upon GNAQ/11 activation. FR900359 is a GNAQ/11 inhibitor. **b**,**c**, Box plots of IP3 (**b**) and IP4 (**c**) levels quantified by mass spectrometry 72 h after *INPP5A* depletion. Mean values of all cell lines (*n* indicates number of cell lines per group) shown as relative FC, *n* = 3 (eight cell lines) or *n* = 2 (six cell lines) independent experiments. **d**,**e**, Quantitative analysis of IP3 (**d**) and IP4 (**e**) levels of HEK293A-Cas9 parental or HA-GNAQ-expressing cells 72 h after transduction with indicated sgRNAs. Data are shown as relative FC to control, *n* = 3 independent experiments. **f**, Immunoblot showing levels of IP3R1 following its depletion with two sgRNAs (sg*ITPR1*#1 and sg*ITPR1*#2) in OMM1-Cas9 cells. **g**, Immunoblot showing levels of IP3R3 following its depletion with two sgRNAs (sg*ITPR3*#1 and sg*ITPR3*#2) in 92.1-Cas9 cells. **h**, Growth of OMM1-Cas9 cells depicted as percentage confluence 18 d post-transduction with the indicated sgRNAs, *n* = 2 independent experiments. **i**, Growth of 92.1-Cas9 cells depicted as percentage confluence 14 d post-transduction with the indicated sgRNAs, *n* = 4 independent experiments. **j**, Schematic depiction of the proteasomal degradation of IP3 receptors. **k**, Immunoblot showing levels of ERLIN2 and IP3R3 after *ERLIN2* depletion with two sgRNAs (sg*ERLIN2*#1 and sg*ERLIN2*#2) in the indicated cell lines. **l**, Clonogenic growth of depicted cell lines 18 d after transduction with the indicated sgRNAs. Positive control, *PLK1*, *n* = 3 for 92.1 and Uacc62 and *n* = 2 for Mel202. All data are presented as mean ± s.e.m.; *P* values were determined by one-way ANOVA with multiple comparisons (**b**,**c**,**i**) or two-way ANOVA with multiple comparisons (**d**,**e**). Box plots show 25th to 75th percentiles, the center line depicts the median and whiskers depict minimum and maximum values (**b**,**c**). Western blots were repeated twice and representative experiments are shown. Source data

**Extended Data Fig. 4:**
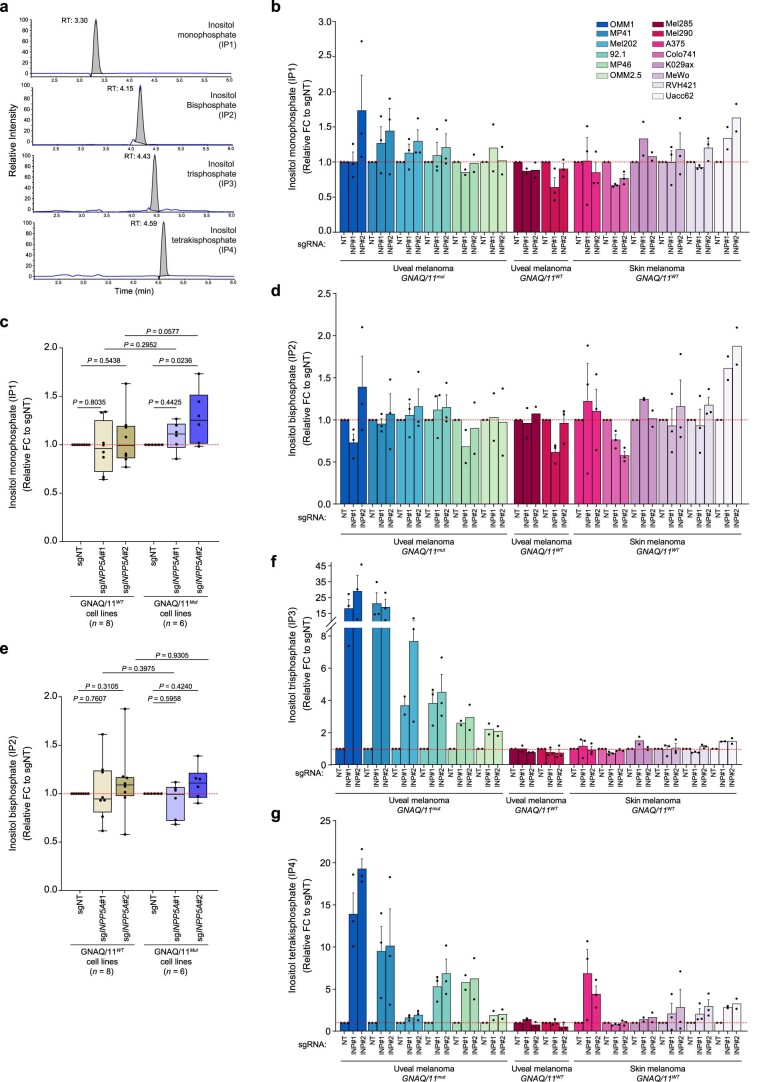
Effect of INPP5A depletion on inositol phosphate levels in uveal and cutaneous melanoma cells. **a**, Representative chromatogram showing retention times of inositol phosphates measured by ion-pair liquid chromatography-tandem mass spectrometry (IP-HPLC-MS/MS). From top to bottom, peaks represent inositol monophosphate (IP1, RT: 3.30), inositol bisphosphate (IP2, RT: 4:15), inositol trisphosphate (IP3, RT: 4:43) and inositol tetrakisphosphate (IP4, RT: 4:59). RT, retention time. **b**, Quantitative analysis of IP1 levels measured by mass spectrometry 72 h after INPP5A depletion depicted for each cell line individually. IP1 levels are shown as fold change relative to negative control (sgNT), *n* = 3 independent experiments for all cell lines except MP46, OMM2.5, Mel285, K029ax and Uacc62 *n* = 2. NT, non-targeting, Mut, mutant, WT, wild-type. **c**, Box plots of the mean values of IP1 levels of all cell lines in (**b**) shown as fold change to relative control (*n* = number of cell lines tested), *n* = 3 independent experiments for 8 cell lines and *n* = 2 independent experiments for 6 cell lines. FC, fold change, NT, non-targeting. **d**, Same as (**b**) for IP2 levels. *n* = 3 independent experiments for all cell lines except MP46, OMM2.5, Mel285, K029ax and Uacc62 *n* = 2. NT, non-targeting, Mut, mutant, WT, wild-type. **e**, Same as (**c**) for IP2 levels. *n* = 3 independent experiments for 8 cell lines and *n* = 2 independent experiments for 6 cell lines. FC, fold change, NT, non-targeting. **f**, Same as (**b**) for IP3 levels. *n* = 3 independent experiments for all cell lines except MP46, OMM2.5, Mel285, K029ax and Uacc62 *n* = 2. NT, non-targeting, Mut, mutant, WT, wild-type. Box plots showing the mean value of all cell lines are shown in Fig. 3b. **g**, Same as (**b**) for IP4 levels. *n* = 3 independent experiments for all cell lines except MP46, OMM2.5, Mel285, K029ax and Uacc62 *n* = 2. NT, non-targeting, Mut, mutant, WT, wild-type. Box plots showing the mean value of all cell lines are shown in Fig. 3c. All data are presented as mean ± s.e.m.; *P* values were determined by one-way analysis of variance (ANOVA) with multiple comparisons (**c**, **e**). Box plots show the 25th to 75th percentiles, center line depicts median and whiskers depict minimum and maximum values (**c**, **e**).

**Extended Data Fig. 5:**
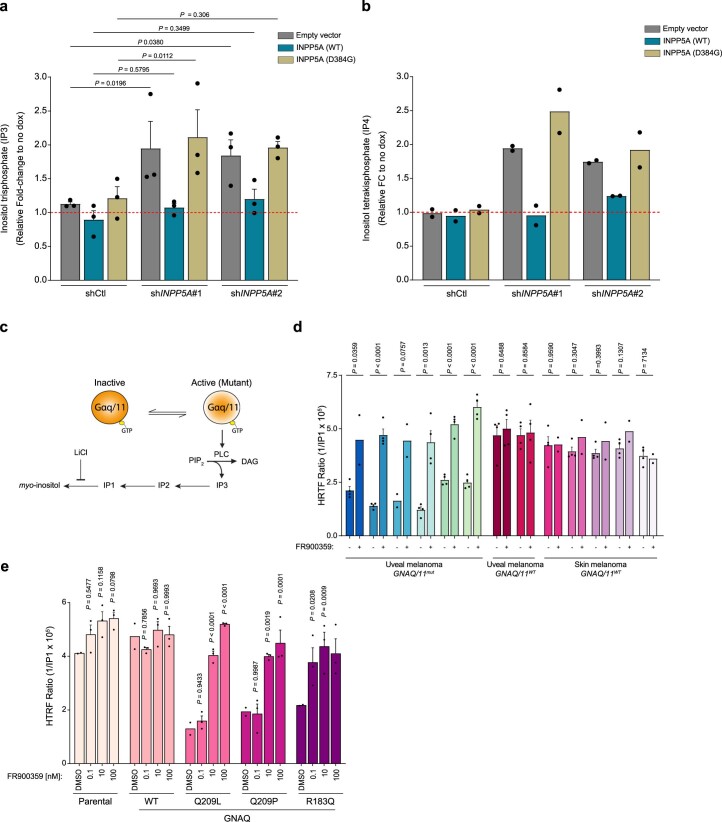
GNAQ/11-mutant cells produce high levels of IP3. **a**, **b**, Quantitative analysis of IP3 (**a**) and IP4 (**b**) levels of 92.1-doxycycline-inducible shRNA cells expressing empty vector or the indicated INPP5A cDNA 72 h after doxycycline treatment. Data are presented as relative fold change to no dox, IP3, *n* = 3 and IP4, *n* = 2 independent experiments. FC, fold change. **c**, Schematic depiction of the metabolic flux of IP3 upon activating the Gα proteins, GNAQ and GNA11. LiCl inhibits the enzymatic activity of inositol monophosphatases (IMPases), leading to the stability of IP1, a downstream metabolite of IP3. IP1 accumulation is used as a surrogate for IP3 synthesis by activated GNAQ/11. **d**, Bar graph showing homologous time-resolved fluorescence (HTRF)-based IP1 accumulation in the indicated cell lines. Ratio of HTRF signal in cells treated with DMSO or GNAQ/11 inhibitor (FR900359 10 nM) for 16 h is shown. HTRF is a competitive immunoassay that measures cellular levels of IP1 which is used as a surrogate for the levels of IP3 produced as described in (**c**). HTRF signal is inversely proportional to the endogenous levels of IP1. GNAQ/11^Mut^ UM cells exhibit enhanced IP3 synthesis driven by their oncogenic mutations compared to GNAQ/11^WT^ cells. Upon inhibition of GNAQ/11 activity by FR, IP3 synthesis is reduced leading to lower accumulation of endogenous IP1 and thus increased HTRF signal, *n* = 4 independent experiments for DMSO and *n* = 2 for FR data for all cell lines except MP41 and 92.1 DMSO *n* = 6, Mel202 DMSO *n* = 3, MP46, OMM2.5, Mel285, Mel290 FR *n* = 4. Mut, mutant, WT, wild-type. **e**, Bar graph showing HTRF-based IP1 accumulation in HEK293A-Cas9 parental cells or cells expressing the indicated HA-GNAQ cDNAs. Cells were treated with DMSO or increasing concentrations of the GNAQ/11 inhibitor (FR900359) for 16 h. An increase in HTRF signal, which is indicative of reduced IP3 synthesis, was achieved at lower FR concentrations in cells expressing GNAQ-R183Q compared to GNAQ-Q209L or GNAQ-Q209P -expressing cells, suggesting that R183Q mutation results in intrinsically less active mutant GNAQ/11 proteins. DMSO, *n* = 2, FR, *n* = 3 biologically independent samples. WT, wild-type. All data are presented as mean ± s.e.m.; *P* values were determined by two-way analysis of variance (ANOVA) with multiple comparisons (**a**, **e**) or two-tailed, unpaired Student’s *t*-test (**d**). Source data

We then sought to delineate the mechanistic basis of INPP5A dependence in GNAQ/11^Mut^ cells. INPP5A hydrolyzes IP3 and IP4 to inositol 1,4 bisphosphate (IP2) and inositol 1,3,4 trisphosphate, respectively (Fig. 3a)^27^. IP3 is a second messenger generated by PLC through the activation of GNAQ/11 (Fig. 3a). We optimized an ion-pair chromatography/mass spectrometry method to dissect changes in intracellular levels of inositol phosphates upon INPP5A inactivation (Extended Data Fig. 4a and Supplementary Table 4)^28^. *INPP5A* knockout did not result in noticeable alterations in inositol monophosphate (IP1) or IP2 levels in wild-type or mutant GNAQ/11 cells (Extended Data Fig. 4b–e). In contrast, depleting INPP5A led to a substantial accumulation of IP3 and IP4 in GNAQ/11^Mut^ UM cells, whereas it hardly affected their levels in GNAQ/11^WT^ UM or CM cells (Fig. 3b,c and Extended Data Fig. 4f,g). Consistently, we observed a marked elevation of IP3 and IP4 in GNAQ^Q209L^-expressing but not in GNAQ^WT^-expressing or parental HEK293A cells upon INPP5A loss (Fig. 3d,e). Importantly, IP3 and IP4 levels were restored by re-expressing wild-type but not phosphatase-deficient INPP5A, suggesting that INPP5A-mediated dephosphorylation is the principal route for their metabolism in GNAQ/11^Mut^ UM cells (Extended Data Fig. 5a,b).

We then probed whether the preferential accumulation of IP3 and IP4 in INPP5A-depleted mutant cells is the outcome of the constitutive signaling of the GNAQ/11 oncoproteins. To test this, we measured GNAQ/11 signaling output by quantifying the accumulation of IP1, a downstream metabolite of IP3 that is stabilized in the presence of LiCl, as a surrogate for IP3 production (Extended Data Fig. 5c)^29^. GNAQ/11^Mut^ cells (UM or HEK293A-GNAQ^Q209L/P or R183Q^) exhibited higher accumulation of IP1 compared to GNAQ/11^WT^ UM, CM or HEK293A cells, demonstrating that the mutant cells produce more IP3 than their wild-type counterparts (Extended Data Fig. 5d,e). Furthermore, inhibiting GNAQ/11 activity with FR significantly suppressed IP3 production and, most importantly, restored the elevated levels of IP3 and IP4 in INPP5A-depleted GNAQ/11^Mut^ cells (Fig. 3d,e and Extended Data Fig. 5d,e). This suggests that IP3 and IP4 preferential accumulation in INPP5A-depleted GNAQ/11^Mut^ cells is driven by the enhanced IP3 synthesis in these cells.

**Extended Data Fig. 6:**
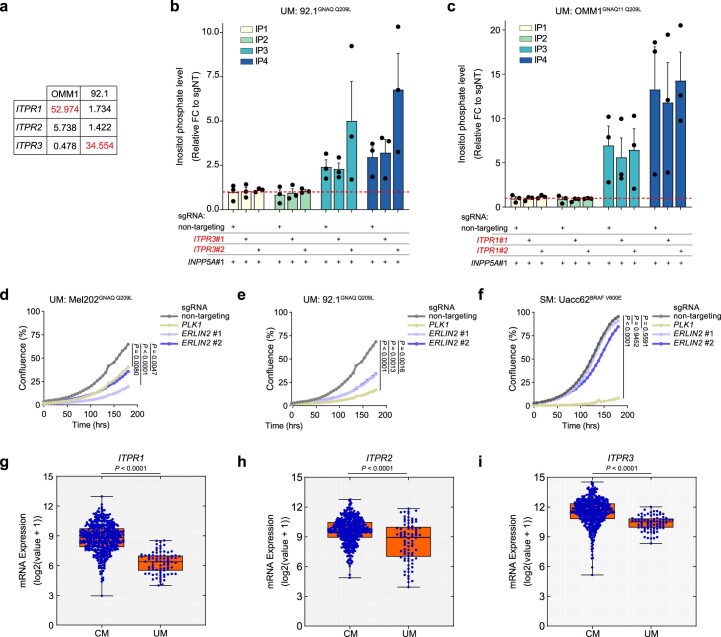
Tight regulation of IP3 receptors is crucial for GNAQ/11-mutant UM cells survival. **a**, Table summarizing CCLE mRNA expression (TPM values) data of *ITPR1*, *ITPR2* and *ITPR3* (encoding IP3R1, IP3R2 and IP3R3 subtypes, respectively) in OMM1 and 92.1 cells. IP3-receptor subtype with the highest expression in each cell line is shown in red. **b**,**c**, Levels of inositol phosphates (IP1, IP2, IP3 and IP4) in 92.1-Cas9 (**b**) and OMM1-Cas9 (**c**) cells expressing the indicated sgRNAs. Cells transduced with sg*ITPR3*#1 or sg*ITPR3*#2 (92.1-Cas9) and sg*ITPR1*#1 or sg*ITPR1*#2 (OMM1-Cas9) were co-transduced with sg*INPP5A*#1 or non-targeting sgRNA and harvested 72 h later. Results are shown relative to NT control sgRNA and dashed line depicts equivalent levels to control, *n* = 3 independent experiments. NT, non-targeting. **d**, **e**, **f**, Growth of Mel202-Cas9 (**d**), 92.1-Cas9 (**e**) and Uacc62-Cas9 (**f**) cells determined by Incucyte live-cell analysis following the transduction with the indicated sgRNAs. *ERLIN2* is depleted with two independent sgRNAs (sg*ERLIN2*#1 and #2), sgNT and sg*PLK1* are negative and positive control sgRNAs, respectively, *n*= 2 independent experiments. NT, non-targeting. **g**, **h**, **i**, Box plots showing TCGA mRNA expression data (TPM values) of *ITPR1* (encoding IP3R subtype 1) (**g**), *ITPR2* (encoding IP3R subtype 2) (**h**) and *ITPR3* (encoding IP3R subtype 3) (**i**) in human UM and CM tumors. UM, uveal melanoma; CM, cutaneous melanoma. All data are presented as mean ± s.e.m.; *P* values were determined by two-way analysis of variance (ANOVA) with multiple comparisons (**b**, **c**), one-way ANOVA with multiple comparisons (**d**, **e**, **f**), or two-tailed, unpaired Student’s *t*-test (**g**, **h**, **i**). Box plots show 25th to 75th percentiles, center line depicts median, and whiskers depict minimum and maximum values (**g**, **h**, **i**). Source data

Because the key function of IP3 is to bind and activate IP3Rs to mobilize calcium, we hypothesized that IP3 accumulation and the consequent IP3 receptors (IP3Rs) activation might mediate the toxic effects of INPP5A depletion in GNAQ/11^Mut^ cells. To test this hypothesis, we first assessed the impact of CRISPR-mediated co-depletion of INPP5A and IP3R on the mutant cells. Mammalian cells express three distinct IP3R subtypes at variable levels^30^. We performed our experiments on two independent UM cell lines, each predominantly expressing a distinct IP3R subtype (Extended Data Fig. 6a). Of note, depleting IP3R1 or IP3R3 markedly rescued the growth inhibition induced by INPP5A loss in OMM1^GNA11-Q209L^ or 92.1^GNAQ-Q209L^ UM cells, respectively, despite the sustained accumulation of IP3 and IP4 (Fig. 3f–i and Extended Data Fig. 6b,c). To further substantiate the notion that efficient termination of IP3 signaling is critical for GNAQ/11^Mut^ UM cells, we depleted ERLIN2, a member of the endoplasmic reticulum (ER)-associated degradation (ERAD) pathway that is responsible for degrading IP3Rs (Fig. 3j)^31^. ERLIN2 loss resulted in the upregulation of IP3R3 levels in all cells irrespective of their genotype and lineage (Fig. 3k); however, its depletion selectively impaired the viability of 92.1 and Mel202 GNAQ^Mut^ UM cell lines but did not affect Uacc62 GNAQ/11^WT^ CM cells (Fig. 3l and Extended Data Fig. 6d–f). In line with these results, analysis of TCGA expression data revealed that all three IP3R subtypes are expressed at lower levels in UM tumors compared to CM, supporting our hypothesis that tight regulation of IP3 signaling is crucial for the survival of GNAQ/11^Mut^ UM cells (Extended Data Fig. 6g–i). In summary, these results suggest that the enhanced synthesis of IP3 renders GNAQ/11^Mut^ UM cells sensitive to INPP5A inhibition by promoting the accumulation of IP3 and aberrant activation of IP3R signaling.

### INPP5A loss increases cytosolic calcium in GNAQ/11-mutant UM

**Fig. 4:**
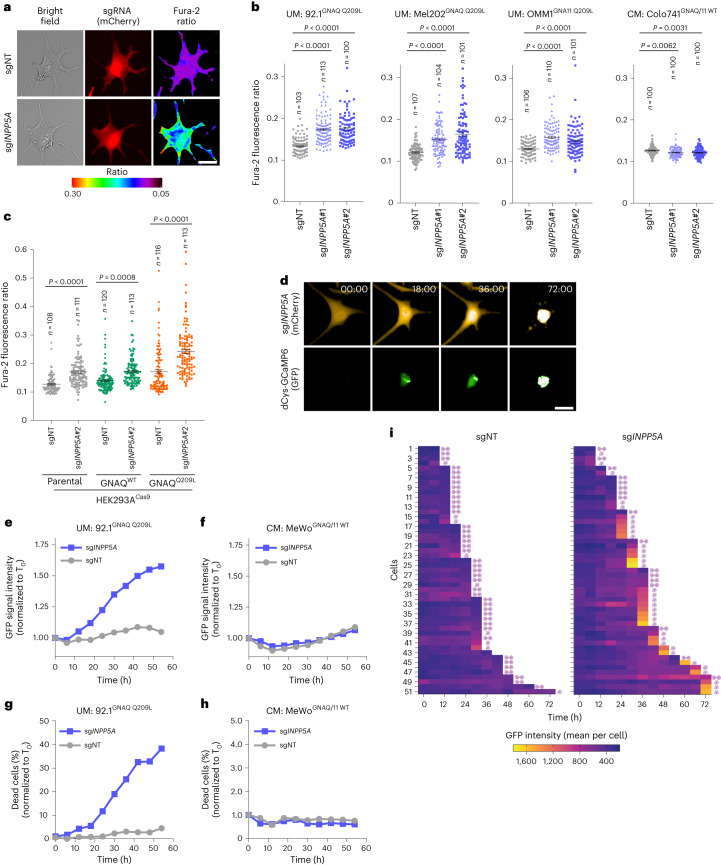
INPP5A depletion preferentially results in elevated cytosolic calcium levels in GNAQ/11-mutant UM cells. **a**, Representative images of the fluorescence signal ratio (F_340_/F_380_) of the ratiometric calcium indicator fura-2 in cells transduced with indicated sgRNA (mCherry^+^). Scale bar, 20 µm. **b**, Quantification of fura-2 fluorescence signal ratio (F_340_/F_380_) 72 h after transduction with the indicated sgRNAs in the depicted cell lines. Each point represents a cell (*n* indicates cell number quantified per group). A representative of *n* = 3 for 92.1 and OMM1 and *n* = 2 for Mel202 and Colo741 independent experiments is shown. **c**, Same as **b** for HEK293A-Cas9 parental or cells HA-GNAQ-expressing cells. A representative of *n* = 2 independent experiments is shown. **d**, Representative time-lapse images of 92.1-Cas9 cells expressing sgRNA (mCherry^+^) and calcium indicator dCys-GCaMP6 (GFP^+^). GFP is activated upon calcium binding to dCys-GCaMP6. Time is depicted in hours. Scale bar, 20 µm. **e**,**f**, Quantification of normalized GFP signal intensity of 92.1-Cas9 (**e**) and MeWo-Cas9 (**f**) cells transduced with the indicated sgRNAs. Cells were treated with the calcium ATPase inhibitor (thapsigargin 1.5 nM) at the start of imaging. A representative of *n* = 2 independent experiments is shown. **g**,**h**, Quantification of the percentage of dead cells of 92.1-Cas9 (**g**) and MeWo-Cas9 (**h**) cells transduced with the indicated sgRNAs and treated with the calcium ATPase inhibitor (thapsigargin 1.5 nM) at the start of imaging. A representative of *n* = 2 independent experiments is shown. **i**, Single-cell-tracking analysis of GFP intensity coupled to cell fate of 92.1-Cas9 cells transduced with sgNT (left) or sg*INPP5A* (right). All cells that either died or divided in randomly selected fields of view were quantified. Each row represents one single cell. A representative of *n* = 2 experiments is shown. All data are presented as mean ± s.e.m.; *P* values were determined by one-way ANOVA with multiple comparisons (**b**,**c**). Source data

**Extended Data Fig. 7:**
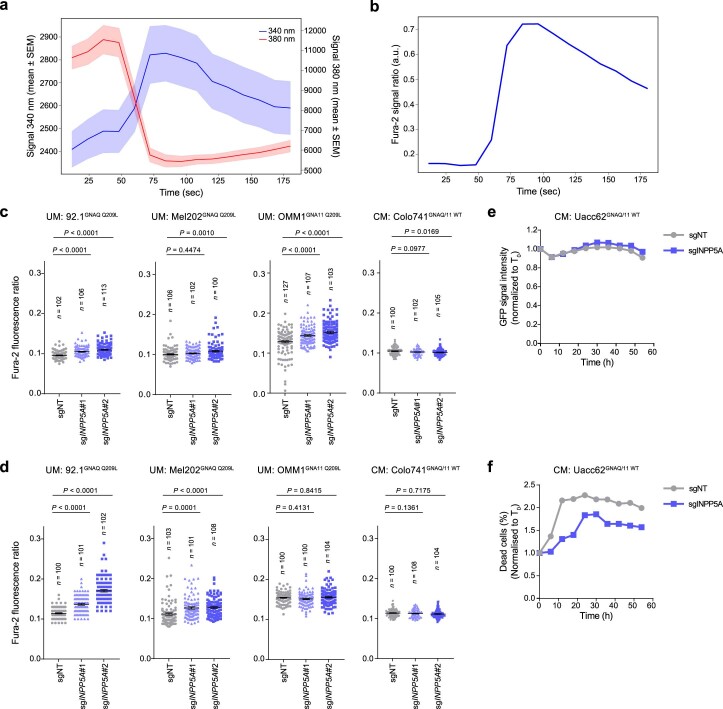
INPP5A depletion preferentially results in elevated cytosolic calcium levels in GNAQ/11-mutant UM cells. **a**, Cumulative time course illustrating the response of fura-2 signal to changes in intracellular calcium levels. The curves depict fura-2 fluorescence signal measured at excitation wavelength of 340 nm (left *y* axis) and 380 nm (right *y* axis). Ionomycin (2 µM) was added to cell culture medium at ~40 seconds after the start of imaging to induce changes in cytosolic calcium levels. **b**, Cumulative time course illustrating intensities of fura-2 fluorescence ratio (F_340_/F_380_) produced by excitation at two wavelengths (340 nm and 380 nm). The increase in cytosolic calcium levels results in an increased ratio of ion-bound indicator (measured at 340 nm) over ion-free indicator (measured at 380 nm). **c, d**, Quantification of fura-2 fluorescence signal ratio (F_340_/F_380_) 24 h (**c**) and 48 h (**d**) after transduction with the indicated sgRNAs in the depicted cell lines. Each point represents a cell; *n* indicates the cell number used for quantification in each group. A representative of *n* = 3 for 92.1 and OMM1 and *n* = 2 for Mel202 and Colo741 independent experiments is shown. **e**, Quantification of normalized GFP signal intensity of Uacc62-Cas9 cells transduced with the indicated sgRNAs. The calcium ATPase inhibitor Thapsigargin (1.5 nM) was added before the start of imaging. Measured signal represents median per well, *n* = 2 independent experiments. **f**, Quantification of the percentage of dead cells of Uacc62-Cas9 cells transduced with the indicated sgRNAs and treated as shown in (**e**), *n* = 2 independent experiments. All data are presented as mean ± s.e.m.; *P* values were determined by one-way analysis of variance (ANOVA) with multiple comparisons (**c**, **d**). Source data

Previous work has shown that INPP5A suppression perturbs calcium homeostasis^32^. To evaluate the effect of INPP5A depletion on calcium signaling in GNAQ/11^Mut^ UM, we employed ratio-imaging microscopy using the high-affinity fluorescent calcium indicator fura-2 (Fig. 4a and Extended Data Fig. 7a,b). Using single-cell calcium measurements following fura-2 loading, we found that INPP5A depletion led to an increase in cytosolic calcium levels in GNAQ/11^Mut^ UM cells and in GNAQ/11^Q209L^-expressing HEK293A cells (Fig. 4b,c and Extended Data Fig. 7c,d). Consistent with previous reports, we observed a minimal but significant increase in calcium levels of GNAQ/11^WT^-expressing and parental HEK293A cells following *INPP5A* knockout (Fig. 4c)^32,33^. In contrast, we did not detect any noticeable alterations in calcium levels in GNAQ/11^WT^ CM cells upon INPP5A depletion (Fig. 4b and Extended Data Fig. 7c,d).

It is well established that the disruption of intracellular calcium signaling triggers cell death^34^. We thereby investigated whether the lethality induced by INPP5A loss in GNAQ/11^Mut^ cells is the consequence of perturbed calcium signaling. To this end, we engineered UM and CM cell lines with the genetically encoded calcium biosensor, dCys-GCaMP6 (ref. ^35^). Upon calcium binding, dCys-GCaMP6-expressing cells produce GFP signal that allows us to track these cells to determine their fate (Fig. 4d). Using live-cell fluorescent imaging, we detected a progressive elevation of the GFP signal in GNAQ/11^Mut^, but not GNAQ/11^WT^ cells, upon INPP5A depletion, validating our findings using the fura-2 indicator (Fig. 4e,f and Extended Data Fig. 7e). Accordingly, we observed a substantial increase in the percentage of dead cells in GNAQ/11^Mut^, but not GNAQ/11^WT^ cells (Fig. 4g,h and Extended Data Fig. 7f). To associate this increase to changes in calcium levels, we performed single cell-tracking analysis (Fig. 4i). Whereas the majority of control cells underwent cell division, most INPP5A-depleted GNAQ/11^Mut^ cells died shortly after an increase in their GFP signal (Fig. 4i). Altogether, these results suggest that the aberrant activation of IP3/IP3R signal axis in INPP5A-depleted GNAQ/11^Mut^ cells leads to increased levels of cytosolic calcium and subsequent cell death.

### INPP5A loss induces p53-mediated apoptosis in GNAQ-mutant UM

**Fig. 5:**
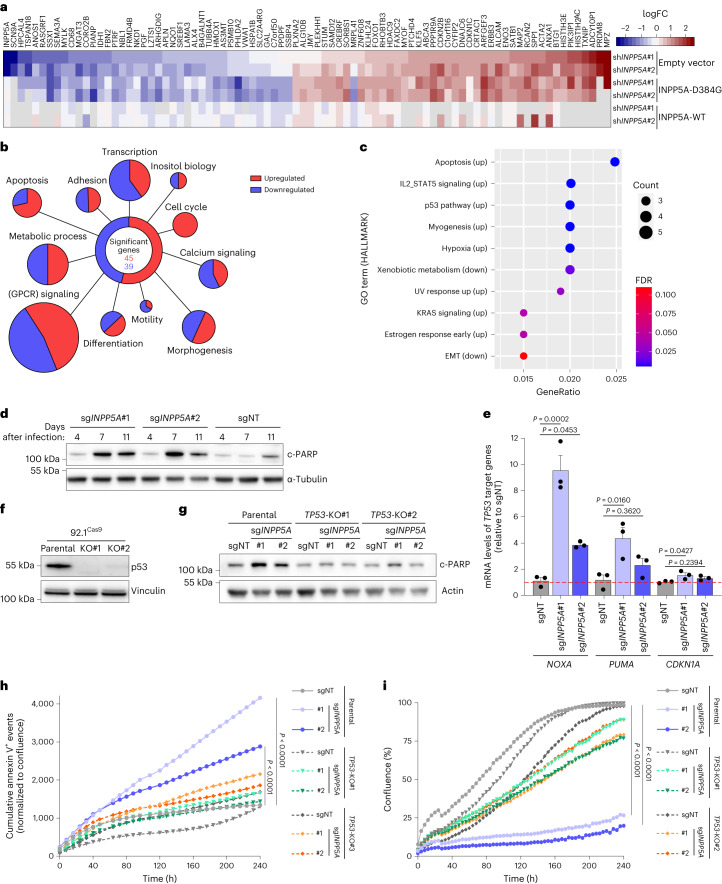
INPP5A depletion induces p53-dependent apoptosis in GNAQ-mutant UM cells. **a**, Heat map of RNA-seq transcriptome analysis showing differentially expressed genes (DEGs; *n* = 84 genes) in 92.1 cells expressing empty expression vector, wild-type or phosphatase-deficient (D384G) INPP5A cDNA. Genes with log_2_-fold change >1 or <−1 in sh*INPP5A*-expressing cells and not with control shRNA are shown (*P* < 0.01, *n* = 3 biological repeats). **b**, Display showing DEGs in **a** categorized per biological function. **c**, Dot plot showing pathways enrichment analysis on HALLMARK gene sets performed on DEGs shown in **a**. Up, upregulated pathways; down, downregulated pathways. **d**, Immunoblot showing levels of c-PARP in 92.1 cells 4, 7 and 11 d after transducing the cells with the indicated sgRNAs. **e**, Quantitative real-time PCR (qRT–PCR) analysis of the p53 target genes *NOXA*, *PUMA* and *CDKN1A* in 92.1-Cas9 cells post-transduction with the indicated sgRNAs, *n* = 3 biologically independent samples. **f**, Immunoblot showing levels of p53 in parental and two independent *TP53* knockout (KO#1 and KO#2) in 92.1-Cas9 cell lines. **g**, Immunoblot showing levels of c-PARP in 92.1-Cas9 parental or *TP53* knockout cell lines transduced with the indicated sgRNAs. **h**, Cumulative annexin V^+^ events normalized to confluence percentage of the depicted cell lines after transduction with the indicated sgRNAs. A representative example of *n* = 3 independent experiments is shown. **i**, Growth curves shown as confluence percentage of the depicted cell lines after transduction with the indicated sgRNAs. A representative example of *n* = 3 independent experiments is shown. Data are presented as mean ± s.e.m.; *P* values were determined by one-way ANOVA with multiple comparisons (**e**,**h**,**i**). Western blots were repeated at least twice with similar results. Source data

**Extended Data Fig. 8:**
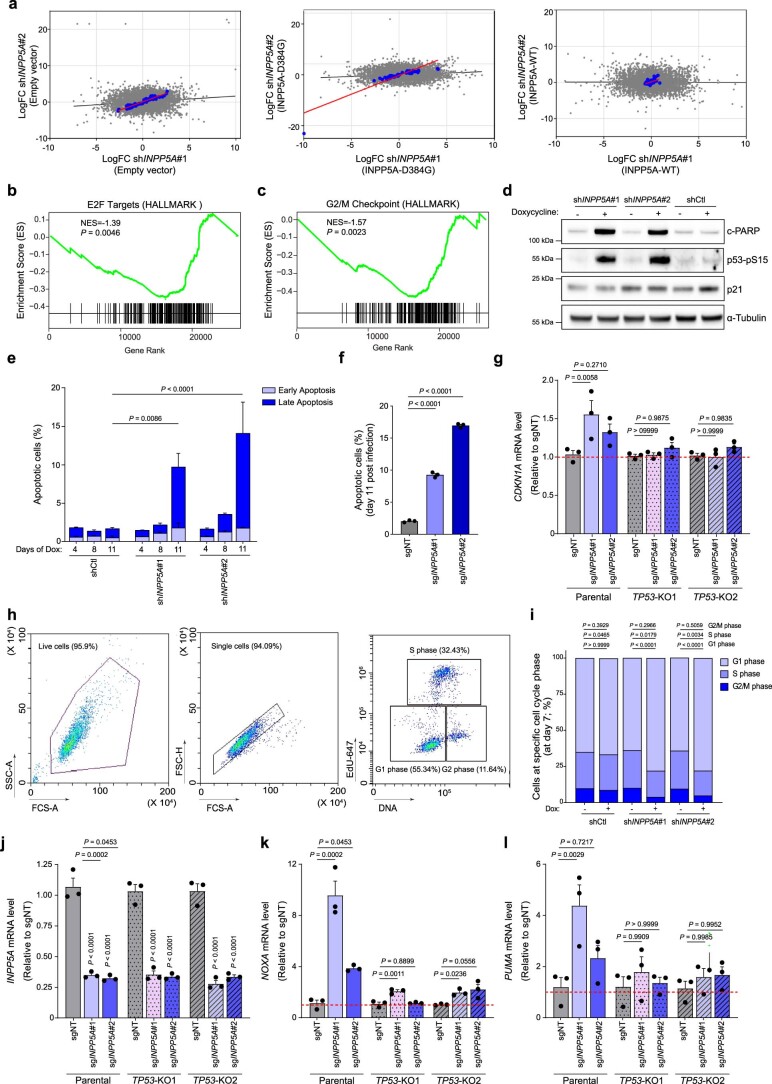
Cellular phenotypes induced by depleting INPP5A in UM cells. **a**, Scatter plots of Log_10_ fold change gene expression as determined by RNA-seq upon depletion of *INPP5A* in 92.1 cell line using two independent sh*INPP5A* (#1 and #2). Cells expressing doxycycline-inducible shRNA and overexpressing empty expression vector (left), phosphatase-deficient INPP5A (INPP5A-D384G) cDNA (middle) or wild-type INPP5A (INPP5A-WT) cDNA (right) were harvested 7 days following doxycycline treatment. Correlations between Log_10_ FC of sh*INPP5A* #1 and sh*INPP5A* #2 are shown for all detected genes (depicted as gray dots and correlation shown as black lines) or only for significant differentially expressed genes (DEGs; *n* = 84, depicted as blue dots and correlation shown as red lines). Profiles of cells transduced with sh*INPP5A*#1 (*x* axis) and sh*INPP5A*#2 (*y* axis) showed good correlation for the DEGs. **b**, **c**, Gene set enrichment analyses (GSEA) signature enrichment plots for E2F targets (**b**) and G2/M checkpoint (**c**) HALLMARK gene sets for the DEGs depicted in (**a**). NES, normalized enrichment score. **d**, Immunoblot showing levels of cleaved PARP (c-PARP), p53-phosphorylated serine 15 (p53-pS15) and p21 in doxycycline-inducible shRNA-expressing 92.1 cells 11 days after doxycycline treatment. α-tubulin is used as loading control. **e**, Stacked bar graph showing percentage of early and late apoptotic cells based on annexin V and propidium iodide staining in doxycycline-inducible shRNA-expressing 92.1 cells at 4, 8 and 11 days after doxycycline treatment. A representative of *n* = 3 independent experiments is shown. Dox, doxycycline. **f**, Bar graph showing percentage of apoptotic cells based on annexin V and propidium iodide staining in 92.1-Cas9 cells at day 11 after transduction with the indicated sgRNAs, *n* = 3 independent experiments. **g**, Quantitative real-time PCR (qRT–PCR) analysis of *CDKN1A* in parental cells and two independent *TP53* knockout (*TP53*-KO1 and *TP53*-KO2) 92.1-Cas9 cell lines after transduction with the indicated sgRNAs, *n* = 3 biologically independent samples. **h**, FACS plots depicting the gating strategy for determining different cell cycle phases. Debris and dead cells were excluded according to the forward scatter-area (FSC-A) and side scatter-area (SSC-A) gating. Singlets were identified based on FSC-A and forward scatter-height (FSC- H) profiles. Then, they were analyzed for propidium iodide (DNA content) and 5-ethynyl-2 deoxyuridine (EdU) coupled to Alexa Fluor 647 (EdU–647) staining intensities. As EdU is incorporated during active DNA synthesis, EdU^+^ cells were classified as ‘S phase’. EdU^−^ cells were classified as ‘G1 phase’ or ‘G2/M phase’ based on their DNA content. **i**, Cell cycle analysis 7 days following shRNA induction with doxycycline in doxycycline-inducible shRNA-expressing 92.1 cells. Percentage of cells at a specific cell cycle phase from total number of live singlet cells is shown. A representative experiment of *n* = 2 independent experiments is shown. **j**, **k**, **l**, Quantitative real-time PCR (qRT–PCR) analysis of *INPP5A* (**j**), *NOXA* (**k**) and *PUMA* (**l**) in parental cells and two independent *TP53* knockout (*TP53*-KO1 and *TP53*-KO2) 92.1-Cas9 cell lines after transduction with the indicated sgRNAs, *n* = 3 biologically independent samples. All data are presented as mean ± s.e.m.; *P* values were determined by two-way analysis of variance (ANOVA) with multiple comparisons (**e**) or one-way ANOVA with multiple comparisons (**f**, **g**, **j**, **k**, **l**). Western blot was repeated twice with similar results. Source data

To gain further insight into the molecular mechanisms underlying the synthetic lethal interaction between INPP5A loss and GNAQ/11 mutations, we performed RNA sequencing upon *INPP5A* silencing (Extended Data Fig. 8a). We knocked down *INPP5A* in 92.1 UM cells and determined the transcriptional changes following the expression of empty vector, wild-type or phosphatase-deficient INPP5A. INPP5A loss induced substantial transcriptional changes in 84 genes (Fig. 5a and Supplementary Table 5). Expression of wild-type but not mutant INPP5A restored the normal transcription of the majority of the affected genes, suggesting that most, if not all, of INPP5A functions require its enzymatic activity (Fig. 5a). Examination of gene ontologies revealed that INPP5A depletion altered the expression of genes associated with GPCR signaling, inositol phosphate biology and calcium regulation, as well as genes involved in key cellular processes such as differentiation, transcription and cell cycle (Fig. 5b). We also performed gene set enrichment analysis (GSEA) of HALLMARK gene sets (Fig. 5c). Notably, p53 and apoptosis pathways were among the significantly upregulated signatures, whereas E2F targets and G2/M checkpoint gene sets were moderately repressed (Fig. 5c and Extended Data Fig. 8b,c).

In line with the transcriptomic data, we found that INPP5A depletion increased the levels of phosphorylated p53 at serine 15, a key post-translational modification required for p53 transcription activity (Extended Data Fig. 8d)^36^. In addition, INPP5A loss in GNAQ^Mut^ UM cells led to an increase of the apoptotic marker cleaved PARP (c-PARP) and the percentage of apoptotic cells as assessed by annexin V staining (Fig. 5d and Extended Data Fig. 8d–f). To pursue the molecular basis underlying this apoptosis, we performed qPCR for the p53 transcription targets, *NOXA*, *PUMA* and *CDKN1A*^37^. INPP5A depletion resulted in significant transcriptional upregulation of both proapoptotic genes, *NOXA* and *PUMA* (Fig. 5e). By contrast, changes in the expression of the cell-cycle regulator, *CDKN1A*, were limited, consistent with the modest increase in G1 cells observed following INPP5A depletion (Fig. 5e and Extended Data Fig. 8d,g–i). Knocking out *TP53* in INPP5A-depleted GNAQ^Mut^ UM cells led to a remarkable rescue of all phenotypic traits, including suppression of the transcriptional upregulation of *NOXA* and *PUMA*, rescue of apoptosis and restoration of normal cell proliferation (Fig. 5f–i and Extended Data Fig. 8j–l). These results suggest that INPP5A depletion in GNAQ/11^Mut^ UM cells prompts a p53-dependent apoptotic response likely as a consequence of the elevation of cytosolic calcium levels.

### INPP5A promotes UM tumor growth and metastasis development

**Fig. 6:**
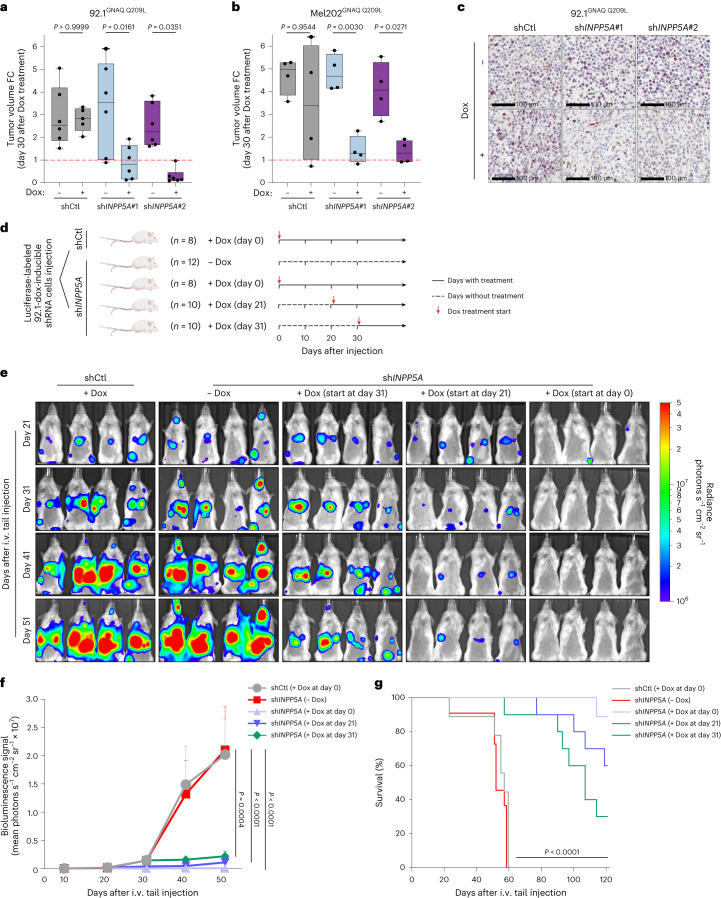
INPP5A is required for UM tumor growth and metastasis development. **a**,**b**, Box plots of tumor volumes of 92.1 (**a**) and Mel202 (**b**) xenografts at day 30 with or without doxycycline treatment shown as relative FC to day 0. Each circle depicts one mouse, for 92.1 xenografts, *n* = 6 mice per group except for the shCtl + Dox group *n* = 5 and for Mel202 xenografts *n* = 4 mice per group. shCtl, control shRNA. Dox, doxycycline. **c**, Immunohistochemistry staining of the proliferation marker Ki67 in 92.1 xenografts with or without Dox induction of the depicted shRNAs at day 10, *n* = 3 mice per group except for the shCtl + Dox group *n* = 2 mice. Scale bar, 100 µm. **d**, Schematic outline for the setup of the in vivo UM metastasis experiment. *n* indicates the number of mice examined per group. **e**, Bioluminescence imaging of NSG mice transplanted with 92.1-luciferase-labeled cells. Cells were transduced with the indicated shRNAs prior transplantation and mice were treated with Dox as indicated. Images were taken on days 21, 31, 41 and 51 post-transplantation. i.v., intravenous. **f**, Quantification of bioluminescence signal from the liver of the mice treated as indicated in **d**. Number of mice used per group is indicated in **d**. **g**, Kaplan–Meier survival curves of mice treated as indicated in **d** until reaching the humane end points (Methods). Data are presented as mean ± s.e.m.; *P* values were determined by two-tailed, unpaired Student’s *t*-test (**a**,**b**) or two-way ANOVA with multiple comparisons (**f**). Survival curves in **g** were assessed with log-rank (Mantel–Cox) test and two-sided Gehan–Breslow–Wilcoxon test. Box plots in **a**,**b** show the 25th to 75th percentiles, the center line depicts the median and whiskers depict minimum and maximum values. Source data

**Extended Data Fig. 9:**
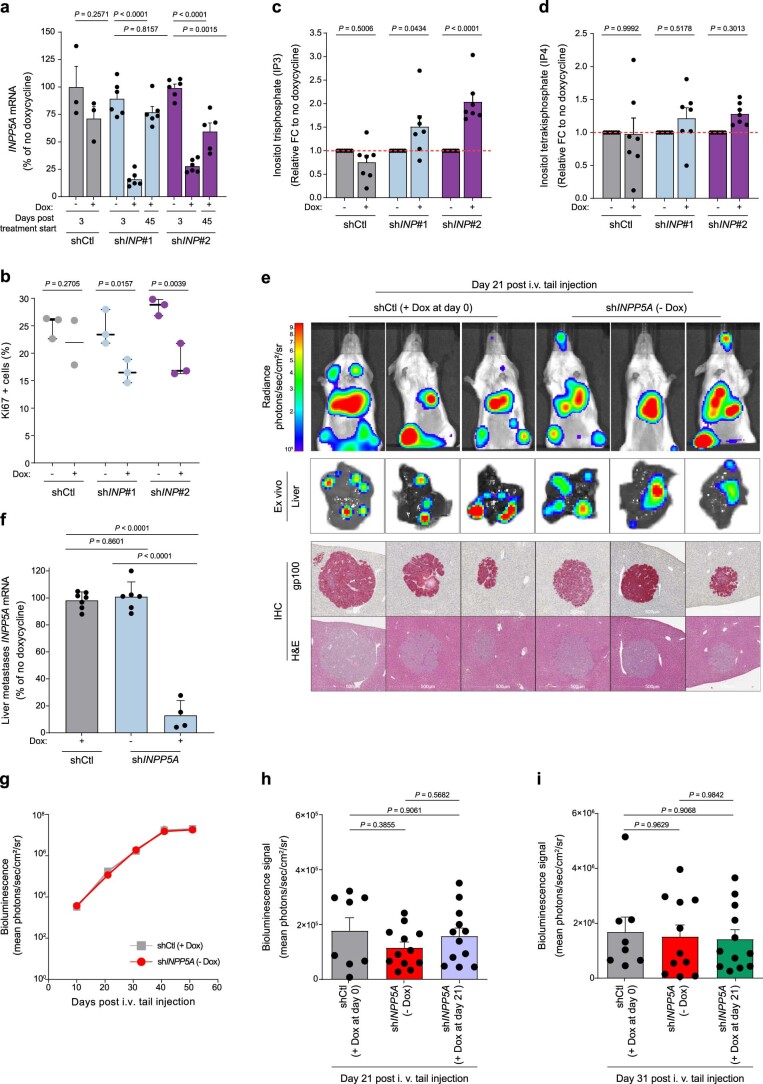
INPP5A is required for UM tumor growth and metastasis development. **a**, Quantitative real-time PCR (qRT–PCR) analysis of *INPP5A* mRNA levels of 92.1 xenografts tumors on day 3 and 45 post doxycycline treatment. Results are shown as percentage of no doxycycline control. shCtl, *n* = 3 mice, sh*INPP5A*#1 and sh*INPP5A*#2 day 3 and 45, *n* = 6 mice, except s*hINPP5A*#2 day 45, *n* = 5. Ctl, control, INP, *INPP5A*. **b**, Quantification of Ki67 immunohistochemistry staining in 92.1 xenograft tumors at day 10 post-induction of the indicated shRNAs; *n* = 3 mice for all conditions, except shCtl + dox, *n* = 2 mice. Ctl, control, INP, *INPP5A*. **c**, **d**, Quantitative analysis of IP3 (**c**) and IP4 (**d**) levels of 92.1 xenograft tumors 72 h after treating the mice with doxycycline (25 mg/Kg q.d.) to induce the expression of the indicated shRNAs. Results are shown relative to no doxycycline control. Dashed line depicts equal levels to control, *n* = 7 mice for all conditions. Ctl, control, INP, *INPP5A*. **e**, Bioluminescence images of NSG mice transplanted with 92.1-luciferase-labeled cells represented at day 21 post-transplantation (top). Cells were transduced with the indicated shRNAs before injection and mice were treated with doxycycline as indicated. Ex vivo bioluminescence images of histological sections of the liver of the mice (middle). The liver signal confirms that injected cells could colonize the liver and form tumor metastases. Immunohistochemistry staining of haematoxylin and eosin (H&E) and the melanocytic marker gp100 confirm that the metastases are originating from the injected UM cells (bottom). Scale bar, 500 µm. **f**, Quantitative real-time PCR (qRT–PCR) analysis of *INPP5A* mRNA levels of liver metastases formed upon transplantation of 92.1-lucifearse-labeled cells transduced with the indicated shRNAs. Results are shown as percentage of no doxycycline, each black dot represents an individual animal, shCtl +Dox and sh*INPP5A* -Dox, *n* = 7, and sh*INPP5A* +Dox, *n* = 4. Data confirm that doxycycline treatment induced the knockdown of *INPP5A* in the liver metastases. **g**, Quantification of bioluminescence signal of the liver of the mice after transplantation of 92.1-luciferase-labeled cells transduced with shCtl or sh*INPP5A*. Images were taken on days 10, 21, 31, 41, 51 and 61 post-transplantation. Data show that doxycycline treatment does not impact the bioluminescence signal and cells transduced with control or *INPP5A* shRNAs have similar growth rates in the liver, *n* = 8 mice per condition. **h**, **i**, Quantification of bioluminescence signal after transplantation of 92.1-luciferase-labeled cells transduced with shCtl or sh*INPP5A* at day 21 (**h**) and day 31 (**i**) post-transplantation. Mice were treated with doxycycline as indicated. Data represent average flux as photons per second for the liver area. The results show that the signal was comparable between control groups (shCtl + dox at day 0 and sh*INPP5A* -Dox) and the treatment groups (sh*INPP5A* + Dox at day 21 or day 31) at the start of the treatment. For treatment group of day 21, shCtl + Dox, *n* = 8, sh*INPP5A* - Dox, *n* = 12, and sh*INPP5A* + Dox, *n* =12, for treatment group of day 31, shCtl + Dox, *n* = 8, sh*INPP5A* - Dox, *n* = 11, and sh*INPP5A* + Dox, *n* =12. All data are presented as mean ± s.e.m.; *P* values were determined by two-tailed, unpaired Student’s *t*-test (**a**, **b**, **f**), two-way analysis of variance (ANOVA) with multiple comparisons (**c**, **d**) or one-way ANOVA with multiple comparisons (**h**, **i**). Source data

We next investigated the impact of INPP5A inhibition on UM tumor growth in vivo. The 92.1 and Mel202 UM cells expressing doxycycline-inducible control and *INPP5A* shRNAs were subcutaneously transplanted into immunocompromised mice treated with doxycycline. Tumor xenografts expressing *INPP5A* shRNAs displayed reduced mRNA levels and significant tumor growth inhibition compared to those harboring a control shRNA (Fig. 6a,b and Extended Data Fig. 9a). The antitumor effect was consistent with a marked reduction in the proliferation marker Ki67 and accumulation of IP3 levels in 92.1 xenografts, suggesting that INPP5A inhibition evokes similar molecular responses in vivo and in vitro (Fig. 6c and Extended Data Fig. 9b–d). Importantly, regrowing tumors have restored their *INPP5A* mRNA levels, further substantiating that UM cells require INPP5A for tumor maintenance in vivo (Extended Data Fig. 9a).

We next determined whether suppression of INPP5A inhibits UM metastasis using a liver metastasis mouse model of UM^38^. Luciferase-labeled UM cells transduced with doxycycline-inducible control or *INPP5A* shRNAs were transplanted into immunodeficient mice (Fig. 6d). Bioluminescent signal, ex vivo imaging and immunohistochemistry staining confirmed the ability of the injected UM cells to form metastatic tumors, predominantly in the liver (Extended Data Fig. 9e). To dissect the role of INPP5A in promoting UM metastasis formation and development, doxycycline treatment was initiated either directly following transplantation or after the formation of the macrometastasis (Fig. 6d, Extended Data Fig. 9f–i). Silencing *INPP5A* substantially inhibited both UM metastasis formation and growth of established metastases, even when treatment onset was delayed until the mice exhibited high metastatic tumor burden (Fig. 6e,f). Moreover, the anti-metastatic effect of INPP5A suppression extended the survival of treated mice as compared to control mice (Fig. 6g). Collectively, these findings demonstrate that INPP5A is required for the development of UM liver metastases.

### IP4 is a predictive biomarker of INPP5A dependence

**Fig. 7:**
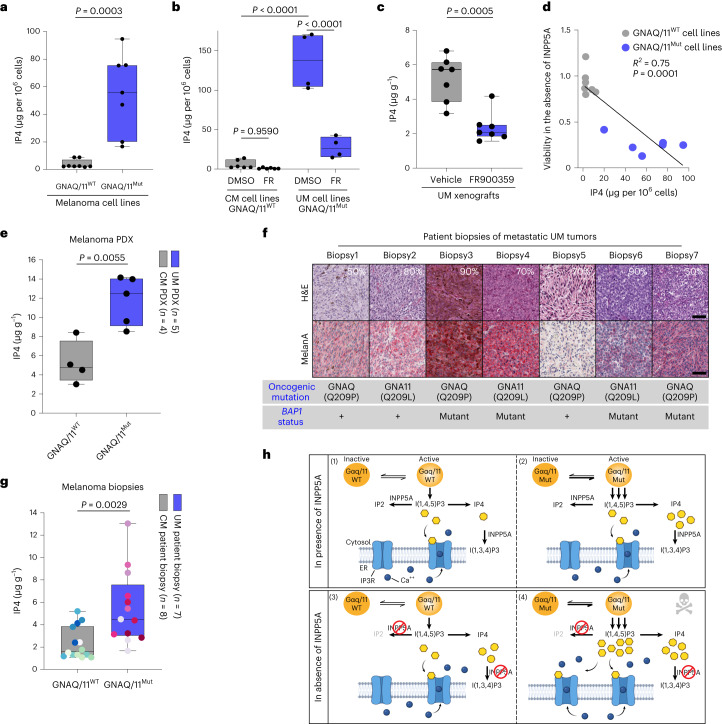
IP4 is a predictive biomarker of INPP5A dependence that is elevated in UM patients’ tumor samples. **a**,**b**, Box plots showing IP4 levels displayed as µg per million cells in GNAQ/11^WT^ cell lines, *n* = 8 and GNAQ/11^Mut^ cell lines, *n* = 7 (**a**) or after treatment with DMSO or GNAQ/11 inhibitor (FR900359 10 nM) for 16 h in GNAQ/11^WT^ cell lines, *n* = 6 and GNAQ/11^Mut^ cell lines, *n* = 4 (**b**). **c**, Box plots showing IP4 levels displayed as µg g^−1^ of tumor tissue of 92.1 xenograft tumors. Mice were treated with vehicle or FR900359 (4 mg kg^−1^, i.v) for 24 h, *n* = 7 mice per group. **d**, Scatter-plot illustrating the correlation between cell viability upon INPP5A depletion and levels of IP4 in µg per million cells in GNAQ/11^WT^ cell lines, *n* = 7 and GNAQ/11^Mut^ cell lines, *n* = 6. **e**, Box plots of IP4 levels shown as µg g^−1^ of tumor tissue of PDX of GNAQ/11^WT^ CM models, *n* = 4 and GNAQ/11^Mut^ or CYSLTR2^Mut^ UM models, *n* = 5. **f**, Representative images of hematoxylin and eosin (H&E) and Melan A immunohistochemistry of UM patients’ tumor samples, *n* = 7 biopsies. Percentage of tumor content, oncogenic mutation and *BAP1* mutational status are shown. Scale bar, 50 μm. **g**, Box plots of IP4 levels shown as µg g^−1^ of tumor tissue of GNAQ/11^WT^ CM patients’ samples, *n* = 8 and GNAQ/11^Mut^ UM patients’ samples, *n* = 7. All biopsies are represented by two distinct histological sections (indicated as matching-color dots), except two biopsies per tumor type each is represented by one section. **h**, Schematic model describing the mechanism of INPP5A dependency in GNAQ/11^Mut^ UM cells. Gαq/11, GNAQ/11 proteins; Ca^++^, calcium. All data are presented as mean ± s.e.m.; *P* values were determined by two-tailed, unpaired Student’s *t*-test (**a**–**c**,**e**,**g**). Two-tailed Pearson’s correlation with 95% confidence interval (**d**). Box plots show the 25th to 75th percentiles, the center line depicts the median and whiskers depict minimum and maximum values (**a**–**c**,**e**,**g**). Source data

**Extended Data Fig. 10:**
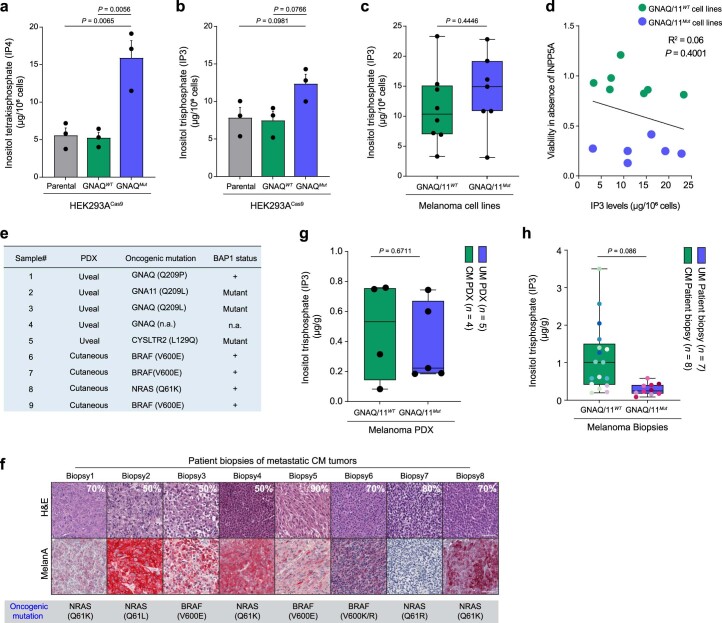
Steady-state levels of inositol phosphates in UM cell lines, patient-derived xenografts, and patient tumor biopsies. **a**, **b**, Graphs showing steady-state levels of IP4 (**a**) and IP3 (**b**) displayed as µg per million cells in HEK293A-Cas9 parental cells (gray) or cells expressing wild-type (green) or Q209L mutant (blue) HA-GNAQ cDNAs, *n* = 3 independent experiments. **c**, Box plots showing steady-state levels of IP3 displayed as µg per million cells in GNAQ/11^*WT*^ cell lines (*n* = 8) and GNAQ/11^*Mut*^ cell lines (*n* = 7). Each dot represents an individual cell line. **d**, Scatter-plot illustrating the correlation of between cell viability upon INPP5A depletion (*y* axis) and steady-state levels of IP3 as µg per million cells (*x* axis) for GNAQ/11^*WT*^ cell lines (green, *n* = 7) and GNAQ/11^*Mut*^ cell lines (blue, *n* = 6). **e**, Table summarizing relevant information for the patient-derived xenograft (PDX) samples analyzed. **f**, Representative images of haematoxylin and eosin (H&E) and Melan A immunohistochemistry staining of CM patients’ tumor samples (*n* = 8 biopsies). Percentage of tumor content and oncogenic mutation are depicted for each biopsy. Scale bars, 50 μm. **g**, Box plots of steady-state levels of IP3 shown as µg per gram of tumor tissue of GNAQ/11^*WT*^ CM patient-derived xenografts (PDX) (*n* = 4) and GNAQ/11^*Mut*^ or CYSLTR2^*Mut*^ UM patient-derived xenografts (*n* = 5). **h**, Box plots of steady-state levels of IP3 shown as µg per gram of tumor tissue of GNAQ/11^*WT*^ CM patients’ tumor samples (*n* = 8 biopsies) and GNAQ/11^*Mut*^ UM patients’ tumor samples (*n* =7 biopsies). All biopsies are represented by two distinct histological sections (indicated as matching-color dots), except two biopsies per tumor type each is represented by one section. All data are presented as mean ± s.e.m.; *P* values were determined by one-way analysis of variance (ANOVA) with multiple comparisons (**a**, **b**), two-tailed, unpaired Student’s *t*-test (**c**, **d**, **g**, **h**). Two-tailed Pearson’s correlation with 95% confidence interval (**d**). Box plots show 25^th^ to 75^th^ percentiles, center line depicts median, and whiskers depict minimum and maximum values (**c**, **g**, **h**). Source data

High levels of IP4 accumulate upon exogenous expression of activated GNAQ mutants^39^. We hypothesized that the high steady-state levels of IP4 may be the consequence of enhanced IP3 synthesis and hence a predictive biomarker of sensitivity to INPP5A inhibition. We indeed found that GNAQ/11^Mut^ cells exhibit significantly higher steady-state levels of IP4 in comparison to GNAQ/11^WT^ cells (Fig. 7a and Extended Data Fig. 10a). In contrast, IP3 steady-state levels were comparable among all cell lines, indicating that IP3 metabolism can efficiently counteract the enhanced IP3 synthesis in GNAQ/11^Mut^ cells (Extended Data Fig. 10b,c). Inhibition of GNAQ/11 with FR led to reduced IP4 levels in GNAQ/11^Mut^ UM cells in vitro and in vivo, confirming that these elevated levels are the result of GNAQ/11-mediated enhanced IP3 synthesis (Fig. 7b,c). More notably, steady-state levels of IP4, but not IP3, positively correlated with sensitivity to INPP5A inhibition across all tested models, supporting the notion that IP4 can be used as a predictive biomarker for INPP5A dependence (Fig. 7d and Extended Data Fig. 10d).

To investigate the clinical significance of INPP5A as a therapeutic target, we examined the steady-state levels of IP4 in UM patient-derived xenografts (PDXs) and patients’ tumor samples (Fig. 7e–g and Extended Data Fig. 10e,f). All UM samples originated from metastatic tumors, some of which harbored inactivating mutations in *BAP1*, an event that frequently occurs in metastatic tumors and correlates with poor prognosis^3,21^. Notably, steady-state levels of IP4 were significantly elevated in GNAQ/11^Mut^ UM PDXs and patients’ samples compared to GNAQ/11^WT^ CM samples indicating that patients’ GNAQ/11^Mut^ UM tumors intrinsically exhibit enhanced IP3 synthesis, the fundamental feature driving INPP5A dependence (Fig. 7e,g and Extended Data Fig. 10g,h). These results suggest that INPP5A depletion may act as a clinically relevant vulnerability for GNAQ/11^Mut^ UM.

## Discussion

Identifying oncogene-induced vulnerabilities holds great promise for developing more efficacious and less toxic anticancer treatments^40^. Here, we identified a synthetic lethal interaction between the inositol polyphosphate 5-phosphatase, INPP5A, and activating mutations in *GNAQ*/*11*. These mutations are the oncogenic driver events in over 90% of UM tumors, implying a broad applicability of INPP5A as a potential therapeutic target for UM tumors. Our findings provide key insights into the basis of the observed synthetic lethality. We propose a model in which the constitutive active state of oncogenic mutant GNAQ/11 results in enhanced synthesis of IP3, rendering the mutant cells highly dependent on INPP5A to metabolize this second messenger to terminate its signaling activity. Loss of INPP5A leads to IP3 accumulation, specifically in the mutant setting, resulting in the hyperactivation of IP3/Ca^2+^ signaling that promotes p53-dependent cell death by apoptosis (Fig. 7h). We also demonstrated that levels of IP4, a biomarker for enhanced IP3 synthesis, exquisitely correlate with sensitivity to INPP5A depletion and is elevated in metastatic GNAQ/11^Mut^ UM cell models, PDXs and patients’ tumors, underlying the potential relevance of INPP5A as a therapeutic target in the clinic.

Recent work has shown that other oncogenic alterations found in UM, such as mutations in *CYSLTR2*, a GNAQ/11-linked GPCR and in *PLCβ4*, a downstream effector of GNAQ/11, stimulate the overproduction of IP3 to a similar extent as *GNAQ/11* mutations^29^. Given that IP3 accumulation is a key driver of INPP5A dependence, we speculate that INPP5A may also act as a vulnerability for tumors harboring these other oncogenic mutations. In addition, molecular characterization of TCGA primary UM revealed that mutant GNAQ/11 signaling might be enhanced in tumors at high risk of metastasis^21^. As enhanced GNAQ/11 signaling translates into increased IP3 production, this analysis suggests that highly aggressive UM tumors that typically correlate with poor prognosis may be sensitive to INPP5A inhibition.

IP3 mobilizes calcium by binding to the IP3Rs at the endoplasmic reticulum^41^. It is subjected to two metabolic changes, dephosphorylation by INPP5A and phosphorylation by multiple IP3 kinases (IP3K), producing IP2 and IP4, respectively^27,42^. Neither IP2 nor IP4 directly stimulates IP3R-mediated calcium release, indicating that both metabolic queues terminate IP3 signaling^43,44^. Our findings, however, reveal that in GNAQ/11^Mut^ UM cells the two metabolic pathways are functionally non-redundant. IP3Ks do not compensate for INPP5A loss in mutant UM, pinpointing a dominant role for INPP5A in metabolizing IP3 in these cells. What determines the contribution of these metabolizing enzymes to IP3 flux is still elusive. One plausible factor could be the catalytic kinetics of IP3K and INPP5A. It has been shown that the kinase possesses higher affinity but lower processivity for IP3 than the phosphatase, suggesting that the kinases might act as the primary route for IP3 metabolism, whereas INPP5A is available as a rapid and durable way to get rid of any IP3 surplus^43^. This division of tasks may explain the selective dependency of GNAQ/11^Mut^ UM cells, which produce excessive amounts of IP3, on INPP5A.

In addition to hydrolyzing IP3, INPP5A also metabolizes IP4 (ref. ^45^). Indeed, IP4 accumulated in INPP5A-depleted cells; however, our results suggest that IP4 accumulation is unlikely to play a major role in driving the dependency of GNAQ/11^Mut^ cells on INPP5A as such. First, upon INPP5A loss, we observed IP4 accumulation in a few CM cell lines and in parental and GNAQ^WT^-expressing HEK293A cells; however, none of these cells accumulated IP3 or suffered from loss of viability. Second, although the physiological role of IP4 is poorly understood, it is known that it does not trigger IP3R-mediate calcium release but instead imposes an inhibitory effect on the receptor’s activity^44,46,47^. In our study, however, we showed that INPP5A depletion led to elevated cytosolic calcium preferentially in GNAQ/11^Mut^ UM cells, suggesting that the receptors are hyperactivated under these conditions. Finally, the co-depletion of INPP5A and IP3R rescued the lethal phenotype of INPP5A loss, confirming that the lethality is mediated explicitly through these receptors. Although we cannot formally rule out a contribution of IP4 to this genetic dependency, our data suggest that the lethality incurred by INPP5A depletion in GNAQ/11^Mut^ cells is mainly attributed to the accumulation of IP3.

Phosphatases are valuable therapeutic targets for various diseases, including cancer owing to their roles in regulating multiple vital cellular processes^48^. INPP5A belongs to the inositol 5-phosphatase family, which consists of ten mammalian members, all containing a conserved catalytic domain capable of hydrolyzing (phospho)inositides^49^. INPP5A was found to be lost in cutaneous squamous cell carcinoma, glioblastoma and leukemia, but its precise role in promoting tumorigenicity is yet to be demonstrated^42,50–52^. Developing selective phosphatase inhibitors was deemed challenging owing to the highly charged and conserved nature of the phosphatases’ active sites^53^; however, recent advances in drug discovery approaches allowed the development of several potent inhibitors, a few of which are currently under clinical assessement^54^. For instance, instead of targeting conserved active sites, newly developed inhibitors bind at allosteric sites beyond the catalytic domains, as successfully demonstrated with the recent discovery of the allosteric inhibitors of the phosphatase SHP2 (ref. ^55^). Moreover, selective irreversible covalent inhibitors have proven beneficial to selectively inhibit historically undruggable phosphatases such as the KRAS oncoprotein. Other approaches, such as protein–protein interaction disruptors or targeted protein degradation are also promising strategies, but they require further understanding of INPP5A biology.

The paucity of effective systemic treatments for primary and metastatic UM underscores the dire need to identify actionable drug targets for this lethal disease. Our study combined transformative functional genomics and comprehensive integrative bioinformatics coupled to profound molecular understanding to discover INPP5A as a synthetic lethal vulnerability in GNAQ/11^Mut^ UM cells. Through this powerful platform, we also identified other attractive gene candidates that warrant further investigation. They may provide major conceptual advances in UM biology and offer opportunities to uncover therapeutic targets against UM.

## Methods

### Animal ethics statement

All animal studies were approved by the local ethics committee (Ethikkommisslon Nordwest und Zentralschweiz) and were performed in accordance with the Federal Animal Protection Act and Order. The experiments were carried out according to the Association for Assessment and Accreditation of Laboratory Animal Care International guidelines. Mice were checked for clinical indications, tumor size and body weight as specified in the experimental licenses (BS-2712 and BS-1974). Mice were killed before reaching the approved humane end points of either tumor size limit of 1,500 mm^3^ or body weight loss of 15% or whenever they showed apparent clinical signs of pain. The maximum tumor size/burden was never exceeded in the studies. Source data are provided for all in vivo experiments.

### Patient ethics statement

Patient biopsies from de-identified patients with melanoma were provided by the Department of Dermatology, University Hospital Zurich, Switzerland. Samples were collected and stored by the Biobank after obtaining a written informed consent (Biobank BASEC-NR_2017-00494). The analysis was conducted under the approval of the local ethics committee (Ethikkommisslon Nordwest und Zentralschweiz authorization no. 2021-02243) in accordance with good clinical practice guidelines and the Declaration of Helsinki.

### Cell culture and cell engineering

Cell lines were obtained from ATCC: A375 (CRL-1619), MeWo (HTB-65), MP41 (CRL-3297) and MP46 (CRL-3298), Sigma: MEL202 (13012457), DSMZ: RHV421 (ACC127), Leiden University Medical Center – M.J. Jager: 92.1 (CVCL_8607), OMM1 (CVCL_6939), OMM2.5 (CVCL_C307), Mel285 (CVCL_C303) and Mel290 (CVCL_C304) or CCLE: Colo741 (CVCL_1133), K029ax (CVCL_8784) and UACC62 (CVCL_1780). All cell lines were cultured in RPMI 1640 (BioConcept). HEK293A and HEK293FT (Thermo Fisher) and A375 cells were cultured in DMEM high glucose (BioConcept). The culture medium was supplemented with 10–20% fetal bovine serum (Seradigm), 2 mM l-glutamine, 10 mM HEPES, 1 mM sodium pyruvate, 1% NEAA and 100 U g ml^−1^ penicillin/streptomycin (BioConcept). Tetracycline-free FBS (F2442, Sigma) was used once cells were engineered with doxycycline-inducible constructs. Cas9-expressing cells were generated by the lentiviral delivery of Cas9 followed by selection with 10 µg ml^−1^ blasticidin or neomycin (Invitrogen). Cas9 expression was determined by flow cytometry (Cas9 antibody, 7A9-3A3, 14697, CST). FACS analysis was performed using CytExpert v.2.4 software (Beckman Coulter). All Cas9-expressing cells used were polyclonal, except for the 92.1-Cas9 and Mel202-Cas9 cell lines, which were obtained from a single clone. A375 cells expressed a variant of Cas9 that is coupled to GFP. Cells expressing doxycycline-inducible shRNAs were obtained by lentiviral transduction of pLKO-TET-ON plasmid or a modified version with a U6 promoter and a GFP cassette.

### Lentivirus production and knockout cell line generation

HEK293FT cells were seeded at a density of 5 × 10^6^ in 75-cm^2^ cell culture flasks (Corning). Cells were transfected with 17.5 µg of ready-to-use lentiviral packaging plasmid mix (Cellecta) and 7–10 µg of the indicated constructs using TransIT-293 transfection reagent (Mirus). Viral supernatant was collected 48 and 72 h post-transfection, filtered through 0.45-µm cellulose acetate filters (Millipore) and stored at −80 °C for subsequent use.

*TP53* lenti-CRISPR-Cas9 knockout cell lines were generated using a pair of sgRNAs each targeting a different exon. *TP53*-KO#1 was generated using sg*TP53*#1 and sg*TP53*#2, whereas *TP53*-KO#2 was generated using sg*TP53*#3 and sg*TP53*#4. To generate *INPP5A*, *ITPR1*, *ITPR3* or *ERLIN2* CRISPR knockout cell lines, a single sgRNA targeting an exon region was used. The GNAQ–Q209P construct was purchased from Twist Bioscience and the GNAQ–Q209L and GNAQ–R183Q constructs were generated using the Quickchange Lightning Site-Directed Mutagenesis kit following the manufacturer’s protocol (Agilent Gernomics). pDONR-HA-GNAQ (Origene) was used as a template. The DpnI-treated PCR amplicon was transformed into XL1-blue chemocompetent bacteria and mutations were confirmed by Sanger sequencing. The sequences of oligonucleotides are provided in Supplementary Table 3.

### Genome-wide CRISPR genetic screen

Genome-wide CRISPR genetic screens were performed as described^56^. In brief, Cas9-expressing UM cells were plated in CellSTACK culture chambers (Corning) and infected with the pooled sgRNA lentivirus at a representation of 1,000 cells per sgRNA with a multiplicity of infection of 0.4. Cells were selected with 2 µg ml^−1^ puromycin (GIBCO) for 4 d and the percentage of sgRNA-infected mCherry^+^) cells was assessed by FACS. Selected cells were propagated and collected at the indicated time points for genomic DNA isolation. DNA sequences containing sgRNA templates were amplified by PCR and fragments were purified with the Agencourt AMpute XP beads (Beckman Coulter Life Sciences) for deep sequencing using the Illumina HiSeq 4000 platform.

### Bioinformatic analysis of genetic screens

For data processing of UM CRISPR screens, the total number of sgRNA reads of each sample was counted and scaled according to the library size. Read counts were normalized using the TMM method in the edgeR Bioconductor package v.3.6.2. For publicly available datasets, CRISPR and RNAi screen data were obtained from DepMap v.2020Q4 (refs. ^17,57^). Essentiality and cell line dependency data were downloaded from project Score^14^. Gene-summary log FC for the three UM screens were calculated. Data analysis proceeded along the following steps:

1. Genes with a median logFC of >−0.5 from each of the three UM CRISPR screens were excluded.
2. Genes with a dependency score from project AVANA (CRISPR), project DRIVE (RNAi) or Achilles (RNAi) of <−0.4 were removed.
3. Genes with a priority score or cell line dependency score of >0.4 from project Score were excluded.
4. To filter out melanocyte-lineage essential genes, genes with a median dependency score <−0.5 in CM cell lines were removed and then any gene that has a dependency score of <−0.5 in Mel285 or Mel290 (GNAQ/11^WT^ UM cell lines) were eliminated.
5. *P* values were determined by performing RSA analysis^58^ and were adjusted against multiple testing using an independent hypothesis weighting (IHW) method as described^59^. An adjusted *P* value cutoff of <0.01 was applied.
6. Overlapping genes among the three UM screens were identified as final hits.

### TCGA and CCLE data analysis

mRNA expression data of TCGA was obtained from the GDC portal and CCLE data from the DepMap portal (v.2022Q1)^19,60^. After excluding liquid tumor data, the 5th and 95th percentiles of mRNA expression (TPM values) were calculated for every single gene for all non-UM tumors and median gene expression was computed from UM samples. If the median gene expression in UM was below the fifth percentile gene expression value, we considered it a lowly expressed gene, whereas if it was higher than the 95th percentile, we considered it a highly expressed gene. To exclude melanocyte-specific lineage genes, the median gene expression per tumor lineage was calculated. *z*-scores were computed and genes with a difference in *z* score between UM and CM of less than 0.5 were excluded. Synthetic lethality comparisons presented in Extended Data Fig. 3j were analyzed from DepMap v.2022Q1.

### RNA sequencing

Cells were plated in six-well plates and treated with 100 ng ml^−1^ doxycycline to induce *INPP5A* knockdown. At the indicated time point, cells were collected using 350 µl RLT buffer and lysates were homogenized using QIAshredder spin columns (QIAGEN, 79656). Total RNA was isolated using RNeasy kit according to the manufacturer’s protocol (QIAGEN, 74104). RNA quality and concentration were determined using the Agilent RNA 6000 Nano kit on the Agilent 2100 BioAnalyzer (Agilent). Then, 200 ng RNA was used as input for the library preparation. RNA-seq libraries were generated using the TruSeq RNA Sample Prep kit v.2 and NGS was performed on a HiSeq2500 (Illumina). Gene-level expression estimates were performed using the Pisces algorithm v.0.1.3.1 as described^61^, using Ensembl release 97 gene models. Differential gene expression changes between no doxycycline and doxycycline samples were calculated using DESeq2 v.1.26.0 (ref. ^62^). Common genes between control and INPP5A shRNAs were excluded. Overlapping genes of sh*INPP5A*#1 and sh*INPP5A*#2 were identified and shared genes between the empty vector or wild-type or D384G INPP5A were eliminated. To find significantly altered pathways, GSEA (http://software.broadinstitute.org/gsea/index.jsp) and Fisher’s exact tests were performed with the MSigDB gene set annotations v.7.5.1 using the FGSEA package v.1.22.0 (ref. ^63^).

### Cell growth assays

For clonogenic assays, Cas9-expressing cells were transduced with sgRNAs by lentiviral transduction. At 24 h later, cells were selected with 1 µg ml^−1^ puromycin (GIBCO) and 72 h after transduction, cells were seeded at a density of 1,000–10,000 cells per well in six-well plates depending on the growth rate of each cell line. Cell lines expressing inducible shRNA constructs were treated with DMSO or 100 ng ml^−1^ of doxycycline (Sigma-Aldrich) 16 h after seeding. After 10–21 d, colonies were fixed with 25% glutaraldehyde (Fluka, G0068), stained with 0.5% crystal violet (Sigma, CVC3886) and dissolved with 10% acetic acid for quantification.

For knockdown-rescue experiments, shRNA-resistant cDNAs of wild-type or phosphatase-deficient (D384G) HA-tagged INPP5A were cloned into the pLNCX2 retroviral constructs. Cells expressing the doxycycline-inducible control or *INPP5A* shRNAs were transduced with the retrovirus of the cDNA or empty expression vectors and expression levels were examined by western blotting.

For Incucyte live-cell proliferation assays, indicated cell lines were infected with lentiviral particles of the sgRNAs. At 24 h post-infection, cells were selected with 1–4 µg ml^−1^ puromycin (Gibco) for 48 h and then seeded at a density of 500–4,000 cells per well in 96-well plates (Corning 3904) depending on the growth rate and the experimental design. Cells were imaged every 6 h using the Incucyte S3 platform (Essen Bioscience). Phase-contrast images were analyzed to assess cell proliferation based on confluence using the Incucyte built-in software.

Proliferation assay was used to determine the effect of the GNAQ/11 inhibitor FR900359 (FR) on the proliferation of examined cell lines. Then, 3,000 cells were seeded into 96-well plates and treated with FR at a concentration range of 0.01 to 100 nM using the HP D300e drug dispenser. After incubation for 96 h at 37 °C, cell proliferation was assessed using the resazurin sodium salt dye reduction assay (alamarBlue assay) using a Synergy HT plate reader (BioTek).

For competition-based proliferation assays, Cas9-expressing HEK293A cells were infected with lentiviral particles of the indicated sgRNAs. Three days after infection, cells were collected and 10,000 cells were plated into two 96-well plates (6–8 replicates per condition) and treated with DMSO or FR (10 nM) if applicable. The remaining cells were stored at −20 °C to measure their inositol phosphate levels by mass spectrometry. Six days after infection, baseline levels of sgRNA-expressing cells were determined by measuring the fraction of mCherry^+^ or GFP^+^ cells by flow cytometry (FACS Calibur, BD Biosciences). At the indicated time point, the percentage of mCherry^+^ or GFP^+^ cells relative to baseline was determined.

### Ion-pair chromatography and mass spectrometry

To measure levels of inositol phosphates from cells, 100,000 cells were plated in six-well plates. At the indicated time points, cells were centrifuged at 142*g* at 4 °C for 5 min, pellets were resuspended in 100 µl extraction buffer (1 M perchloric acid with 3 mM EDTA) and stored at −20 °C. For ion-pair high-performance liquid chromatography-tandem mass spectrometry (IP-HPLC–MS/MS), Precellys ceramic beads (1.4 mm, Bertin Instruments, P000927-LYSK0-A) were added before sonication in an ultrasonic bath for 5 min. Then, 60 µl neutralization buffer (1 M Na_2_CO_3_ with 3 mM EDTA) was added and supernatants were collected and transferred to a 0.2-µm Captiva ND 96 filter well plate (Agilent, A5969002) mounted on a vacuum manifold and prewashed with 200 µl ACN:water (1:1, *v*/*v*). Eluates were collected in 96-well plates and 6 µl 2.5 µg ml^−1^ internal standard (IS) was added for normalization.

For tumor samples, isolated tumors were homogenized with twofold (*w*/*v*) ACN:water (2:8, *v*/*v*) and 3 mM EDTA for four cycles of 20 s at 2,460*g* at 4 °C in Precellys tubes (Bertin Instruments, P000916-LYSK0A) using a Precellys 24 Tissue Homogenizer (Bertin Instruments, 000669-PR240-A). Then, 100 µl extraction buffer was added to 20 µl tumor homogenate in protein LoBind tubes and samples were sonicated for 5 min with Precellys ceramic beads (2.8 mm, Bertin Instruments, P000926-LYSK0A). The extraction was repeated twice and supernatants were combined into 1.2 ml 96-deep-well plates to which 120 µl neutralization buffer with 8 µl IS was added. Plates were stored overnight in a precooled auto sampler before the start of the LC–MS analysis. Prepared samples were analyzed using the Nexera X2 HPLC system platform (Shimadzu) coupled to a triple quadrupole mass spectrometer QTRAP 6500+ (Sciex). Data acquisition was performed with Analyst v.1.7 software.

### Quantitative real-time PCR

The 92.1-doxycycline-inducible shRNA cells were plated in six-well plates at a density of 250,000 cells per well. At 72 h after doxycycline treatment (100 ng ml^−1^), mRNA was extracted using QIAshredder spin columns (QIAGEN, 79656) and RNeasy mini kit (QIAGEN, 74106) according to the manufacturer’s protocol. For quantitative PCR reactions, 32 ng mRNA, *GAPDH* probe (Hs.PT.39a.22214836), *INPP5A* probe (Hs00897218_m1) (Integrated DNA Technologies) and iTaq Universal Probes One-Step kit (BioRad, 172-5140) were pipetted in 384-well plates. Quantitative real-time PCR (qRT–PCR) was performed using the probe-based assay with a 7900HT thermal cycler (Applied Biosystems). *GAPDH* mRNA levels were used for normalization and relative expression levels of genes were calculated with the 2^−ΔΔCt^ method. To quantify mRNA levels of *NOXA*, *PUMA* and *CDKN1A*, 20,000 92.1-doxycycline-inducible parental or *TP53* knockout (KO#1 or KO#2) cells were plated in six-well plates. Then, 7 and 11 d after doxycycline treatment, the total RNA was extracted and cDNA synthesis was performed using MultiScribe reverse transcriptase (Thermo Fisher, 4311235). qRT–PCR was performed using FastStart Universal SYBR green master (Roche Rox, 4913850001). Analysis was performed using the Sequence Detection System (SD) v.2.4.1.

### Apoptosis assays

Cells expressing doxycycline-inducible shRNAs were treated with either DMSO or 100 ng ml^−1^ doxycycline for 3 d before seeding in 96-well plates. The medium was refreshed every 3 d. At the indicated time points, cells were collected and apoptosis was determined using the annexin V/Dead Cell Apoptosis kit (Invitrogen, V13245 and A23204) according to the manufacturer’s protocol. In brief, samples were incubated with 100 µl apoptosis reagent (97.5 µl 1× annexin-binding buffer, 0.5 µl 100 µg ml^−1^ propidium iodide and 2 µl Alexa Fluor 647 annexin V) for 15 min on ice. Stained cells were examined using flow cytometry using the CytoFLEX-S analyzer (Beckman Coulter). Untreated or staurosporine-treated cells were used as negative and positive control for apoptosis, respectively.

For live-cell analysis of apoptosis, 92.1-Cas9 parental or *TP53* knockout (KO#1 and KO#2) cells were infected with lentiviral particles of the indicated sgRNAs. Two days after puromycin selection, cells were plated in 96-well plates (Corning, 3904) and treated with Incucyte annexin V dye (1:200 dilution, Sartorius, 4642). Cells were imaged every 4 h using the Incucyte SX3 or SX5 platforms (Essen Bioscience). Red and phase-contrast images were analyzed to quantify red objects (apoptotic events) and percentage of confluence using the Incucyte built-in software. The number of red objects was normalized to confluence percentage and the cumulative count of normalized red objects (obtained by summing up the number of red objects every 8 h) was plotted.

### Cell cycle analysis

For cell cycle analysis, 92.1 cells were seeded in six-well plates. At 16 h post-seeding, DMSO or 100 ng ml^−1^ doxycycline was added to the cells. At the indicated time points, cells were pulsed with 10 µM EdU (Sigma-Aldrich, BCK-FC647) for 1 h before harvesting. Samples were processed according to the manufacturer’s protocol and then incubated with 3 µM 4′,6-diamidino-2-phenylindole for 5 min before imaging by flow cytometry.

### HTRF-based IP1 accumulation assay

To assess IP3 synthesis, accumulation of IP1 was measured as a surrogate of IP3 production using HTRF technology^64^. IP3 is converted to IP1, which is degraded to D-*myo*-inositol. IP1 degradation is inhibited by LiCl, resulting in its accumulation. Endogenous IP1 accumulation leads to a decrease in FRET signal as it competes with exogenously added acceptor-labeled IP1 for the binding to a donor-labeled anti-IP1 antibody (CisBio, 62IPAPEB). Experimentally, 2,500 cells were plated in 96-well Nunclon plate (Corning) and treated overnight with FR900539 (10 nM). Cells were washed, 70 µl stimulation buffer containing LiCl was added and the plate was incubated at 37 °C for 1 h. Then, 15 µl exogenous IP1 and 15 µl antibody-cryptate were added and the plate was incubated for 1 h at room temperature. The readout was performed with a Tecan M1000pro reader.

### Phosphatase activity assay

To assess the phosphatase activity of INPP5A-D384G mutant, 92.1-doxycycline-inducible shRNA cells expressing empty expression vector, wild-type or mutant INPP5A (D384G) cDNAs were plated and treated with 100 ng ml^−1^ doxycycline. At 72 h post-induction, protein samples were extracted in 25 mM TRIS-HCl, 250 mM sucrose, 500 mM KCl, 1 mM dithiothreitol (DTT), 10 mM MgCl_2_ and 2 mM EGTA. Supernatants were collected after centrifuged for 45 min at 16,627*g* at 4 °C. Then, 10 µg protein was incubated with 1 µg inositol 1, 4, 5 trisphosphate (Cayman Chemical) for 2 min and a malachite green phosphate assay kit was used to measure the levels of inorganic free phosphate according to the manufacturer’s protocol (Cayman Chemical, 10009325).

### Microscopy

For cytosolic calcium imaging with fura-2, sgRNA-infected cells were plating in µ-Slide eight-well ibiTreat precoated plates (Ibidi). At the indicated time points, cells were incubated with 5 µM fura-2-AM dye (Invitrogen, F1221) in phenol-red free RPMI medium (BioConcept) for 45 min at 37 °C. Images were acquired on an Olympus inverted IX83 frame equipped with an incubator maintained at 37 °C and 5% CO_2_ (CellVivo, PECON). Images were recorded with an Olympus silicon-immersed ×30 objective. For UV excitation, a X-Cite Exact 200W Hg light source (Excelitas Technologies) was used. Images were acquired using a Hamamatsu Orca Flash 4.0 CCD camera. Then, 2 µM ionomycin (Sigma-Aldrich, I9657-1MG) was used as a positive control. Image analysis and quantification was done using the Olympus cellSens v.3.1.1software.

For live-cell calcium imaging and cell-fate analysis, a dCys-GCaMP6 calcium biosensor was used, which is composed of calmodulin calcium-sensing and M13 peptide-binding elements and a GFP moiety that is activated by binding to calcium. dCys-GCaMP6-expressing 92.1-Cas9, MeWo-Cas9 or Uacc62-Cas9 cells were seeded into 96-well CellCarrierUltra plates (PerkinElmer). At 16 h later, cells were transduced with sgRNAs by lentiviral transduction. At 24 h post-transduction, cells were treated with DMSO or thapsigargin 1.5 nM (Thermo Fisher, T7458) before imaging. Confocal time-lapse images were captured every 6 h for 3 d on an Opera Phenix High Content Imager (PerkinElmer) using ×20/1.0 water objective with sequential exposures for GFP and mCherry. A total of 53 fields were acquired per well and analyzed using the Harmony image analysis software package v.4.9 (PerkinElmer). For analysis, primary cell objects were identified based on mCherry positivity. Mean GFP intensity for each cell was calculated. Single cell values were used to calculate the well median of mean GFP intensity per cell (Spotfire, Tibco). Round cells were classified based on area and roundness thresholds to calculate percentage of round (dead) cells; at least 660 objects per well were counted. For single-cell-tracking analysis, fields were selected randomly and the mean GFP signal intensity was recorded per time frame until the cell either died or divided. Cells that did not change their fate until the end of recording were excluded.

### Western blot

Total cell lysates were incubated with lysis buffer consisting of 50 mM Tris-HCl pH 7.8, 0.01% NP40 (Fluka, 74385), 120 mM NaCl (Fluka, 71380), 25 mM NaF (Merck, 106450), 40 mM β-glycerol phosphate (Fluka, 50020), 100 µM sodium metavanadate (Sigma, 590088), 1 mM DTT (Sigma, 43815), 100 µM phenylmethyl sulfonyl fluoride (Sigma, P7626), 1 mM benzamidine (Sigma, B6506) and 1 µM microcystin (Alexis Biochem, 350012). Protein levels were determined using a Bradford assay (BioRad, 500-0006). Equal protein amounts were loaded into NuPage precast polyacrylamide gels (Invitrogen), transferred to methanol-activated PVDF membranes (Millipore) and 5% milk-blocked membranes were incubated with the following antibodies at 4 °C overnight; GNAQ (1:1,000 dilution, D5V1B, 14373, CST); α-tubulin (1:1,000 dilution, T9026, Sigma-Aldrich); α-actinin (1:1,000 dilution, E7U1O, 69758, CST); ERLIN2 (1:500 dilution, NB100-1884, Novus Biologicals); IP3R3 (1:1,000 dilution, 610312, BD Biosciences); IP3R1 (1:500 dilution, NB120-5908, Novus Biologicals); c-PARP (1:1,000 dilution, 9541, CST); p53 (1:500 dilution, 2527, CST); p21 (1:1,000 dilution, DCS60, 2946, CST); p53-phosphoSer15 (1:1,000 dilution, 9284, CST); HA (1:1,000 dilution, ab9110, Abcam); actin (1:20,000 dilution, C4, MAB1501, Millipore); BAP1 (1:1,000 dilution, D7W70, 13271, CST); pERK (1:1,000 dilution, 9101, CST) and pRASGRP3 (1:1,000 dilution, 124823, Abcam). Membranes were washed with PBS with 0.1% Tween-20 solution and incubated with secondary antibodies; anti-rabbit IgG (1:5,000 dilution, NA931-VS, Amersham); anti-mouse IgG (1:2,500 dilution, 7076, CST) or anti-goat IgG (1:20,000 dilution, A5420, Merck) for 1 h at room temperature. Membranes were imaged using the Fusion FX7 imager (Vilber) after incubation with ECL solution (Amersham, NA931-VS).

### In vivo mouse experiments

Animals were kept under optimal hygiene conditions in Allentown mice cages (maximum of five animals per cage) with a 12-h dark–light conditions and they were fed sterilized food and water ad libitum. For mouse xenograft experiments, naive female athymic nude mice, 6–8 weeks, 20–25 g (Charles River Laboratories) were injected subcutaneously with 5 × 10^6^ of 92.1 or Mel202 cells expressing doxycycline-inducible shRNAs suspended in 200 µl 50% Matrigel (Corning, 354234) and 50% HBSS (Sigma, H6648). Experiments were initiated once the mean size of tumors reached around 200 mm^3^ (13–19 d post-implant) by administering doxycycline (25 mg kg^−1^) daily using oral gavage. Tumors were measured using caliper and tumor volumes were calculated using a modified ellipsoid formula: tumor volume = *L* × *W*^2^ × π/6, where *L* is the longest axis of the tumor and *W* is perpendicular to *L*.

For liver metastasis experiments, 6–8-week-old NOD.Cg-Prkdcscid Il2rgtm1Wjl/SzJ (NSG) female mice (Taconic) were used. Liver metastasis tumors were established by injecting 2 × 10^6^ 92.1 expressing firefly luciferase and doxycycline-inducible shRNAs i.v. via the lateral tail vein. Doxycycline (25 mg kg^−1^) was given daily starting at the indicated time points using oral gavage or in drinking water. For in vivo imaging, mice received d-luciferin (150 mg kg^−1^, 10 ml kg^−1^) through tail vein injections before being anesthetized using isoflurane (2%). Firefly bioluminescence was measured by an In Vivo Imaging System (IVIS) Lumina XR (Xenogen) 6–10 min after luciferin injection using a highly sensitive, cooled CCD camera. Imaging and quantification of signals were conducted by image software Living Image v.4.7.4 (64-bit) (Xenogen). Bioluminescence was measured at days 10, 21, 31, 41, 51 and 61 post-tumor cell injections.

### Histopathology and immunohistochemistry

For patient tumors, the corresponding FFPE blocks of the slow-frozen biopsies were cut (4-µm thickness) and processed as described^65^. Tumor xenograft FFPE blocks were sliced into 3-µm sections and H&E staining and immunohistochemistry were prepared as described^66^. For patient samples, slides were stained with H&E and Melan A (1:200 dilution, Novus, NBP1-30151). Tumor content was evaluated by two independent pathologists. Staining was performed on a Leica BondRX platform (Leica Microsystems). Antibodies were diluted with diluent antibody and alkaline phosphatase chromogen (Leica Bond kit) was used for detection (Bond Polymer Refine Red Detection kit). Ki67 Immunohistochemical staining was performed using Epitope Retrieval 2 (ER2) pretreatment conditions for 20 min at 100 °C. Refine Red kit (Leica Biosystems) was used for detection and the primary antibody was anti-human Ki67 (1:2,000 dilution, SP6, Neomarker, RM9106). Slides were then hematoxylin counterstained, dehydrated, cover-slipped and digitalized with Aperio AT2. Ki67 quantification was conducted using the HALO image analysis platform (v.3.1.1076.437) (Indica Labs) CytoNuclear IHC algorithm.

### Patient-derived xenografts

UM PDXs were purchased as snap-frozen samples from Xenostart and Charles River laboratories. CM PDXs and one UM PDX were grown in-house and are the property of the Novartis Institute for Biomedical Research.

### Statistics and reproducibility

Data are presented as indicated in the respective figure legends. In vitro experiments were performed in biological triplicate or duplicate. No statistical method was employed to predetermine sample size. Data collection and analysis were not performed blind to the conditions of the experiments. Data distribution was assumed to be normal, but this was not formally tested. For in vivo experiments, mice were randomized into control and treatment groups within their genotype, while maintaining comparable tumor volume averages per group. No data were excluded from the analysis, except for Fig. 6a, where one mouse was excluded as it did not develop a tumor, and Fig. 6d, when one mouse died under anesthesia. A few patient biopsies were excluded as they failed to meet the quality control criteria (Reporting Summary provides details).

All data are presented as mean ± s.e.m. No assumptions on data distribution were made. Statistical analysis was performed with GraphPad Prism 9 software using a two-tailed, unpaired Student’s *t*-test, one-way ANOVA, two-way ANOVA or multiple Student’s *t*-test. Corrections for multiple comparisons were performed according to the recommended method by the software. Correlation analysis was performed using Pearson’s correlation test and *R*^2^ values are displayed. *P* values of <0.05 were considered statistically significant for all experiments. Box plots show the 25th to 75th percentiles, the center line depicts the median and whiskers depict minimum and maximum values.

### Reporting summary

Further information on research design is available in the Nature Portfolio Reporting Summary linked to this article.

### Supplementary information

Reporting Summary

Supplementary Table Supplementary Tables 1–5

### Source data

Source Data Fig. 1 Statistical source data.

Source Data Fig. 2 Statistical source data.

Source Data Fig. 2 Unprocessed western blots.

Source Data Fig. 3 Statistical source data.

Source Data Fig. 3 Unprocessed western blots.

Source Data Fig. 4 Statistical source data.

Source Data Fig. 5 Statistical source data.

Source Data Fig. 5 Unprocessed western blots.

Source Data Fig. 6 Statistical source data.

Source Data Fig. 7 Statistical source data.

Source Data Extended Data Fig. 1 Statistical source data.

Source Data Extended Data Fig. 2 Statistical source data.

Source Data Extended Data Fig. 2 Unprocessed western blots.

Source Data Extended Data Fig. 3 Statistical source data.

Source Data Extended Data Fig. 3 Unprocessed western blots.

Source Data Extended Data Fig. 4 Statistical source data.

Source Data Extended Data Fig. 5 Statistical source data.

Source Data Extended Data Fig. 6 Statistical source data.

Source Data Extended Data Fig. 7 Statistical source data.

Source Data Extended Data Fig. 8 Statistical source data.

Source Data Extended Data Fig. 8 Unprocessed western blots.

Source Data Extended Data Fig. 9 Statistical source data.

Source Data Extended Data Fig. 10 Statistical source data.

## Data Availability

RNA-seq data presented in this study were deposited into the Sequence Read Archive under accession no. PRJNA860930. RNA expression data derived from TCGA data were downloaded from the GDC data portal (GDC (cancer.gov)). Cancer cell line dependency and RNA expression data were downloaded from DepMap (DepMap: The Cancer Dependency Map Project at Broad Institute) and internal RNA-seq datasets. UM CRISPR screen data are shown in Supplementary Table 1. All other data that support the findings of this study can be made available from the corresponding author upon request. Source data are provided with this paper.

## Code availability

No custom algorithms were used in this study. Open-source software was used to analyze data. Details of software versions are specified in the Methods.
