# Supplementary material for: INPP5A phosphatase is a synthetic lethal target in GNAQ and GNA11-mutant melanomas
Source: Nat Cancer. 2024 Jan 17;5(3):481–99. doi: 10.1038/s43018-023-00710-z (PMC10965444; doi:10.1038/s43018-023-00710-z)
Supplement: Supplementary file 1 — Reporting Summary [file 43018_2023_710_MOESM1_ESM.pdf]

nature portfolio

Corresponding author(s): Eusebio Manchado

Last updated by author(s): Nov 23, 2023

Reporting Summary

Nature Portfolio wishes to improve the reproducibility of the work that we publish. This form provides structure for consistency and transparency in reporting. For further information on Nature Portfolio policies, see our Editorial Policies and the Editorial Policy Checklist.

Statistics

For all statistical analyses, confirm that the following items are present in the figure legend, table legend, main text, or Methods section.

n/a	Confirmed	
☐	☒	The exact sample size (*n*) for each experimental group/condition, given as a discrete number and unit of measurement
☐	☒	A statement on whether measurements were taken from distinct samples or whether the same sample was measured repeatedly
☐	☒	The statistical test(s) used AND whether they are one- or two-sided<br>*Only common tests should be described solely by name; describe more complex techniques in the Methods section.*
☒	☐	A description of all covariates tested
☐	☒	A description of any assumptions or corrections, such as tests of normality and adjustment for multiple comparisons
☐	☒	A full description of the statistical parameters including central tendency (e.g. means) or other basic estimates (e.g. regression coefficient) AND variation (e.g. standard deviation) or associated estimates of uncertainty (e.g. confidence intervals)
☐	☒	For null hypothesis testing, the test statistic (e.g. F, t, r) with confidence intervals, effect sizes, degrees of freedom and P value noted<br>*Give P values as exact values whenever suitable.*
☒	☐	For Bayesian analysis, information on the choice of priors and Markov chain Monte Carlo settings
☐	☒	For hierarchical and complex designs, identification of the appropriate level for tests and full reporting of outcomes
☐	☒	Estimates of effect sizes (e.g. Cohen's *d*, Pearson's *r*), indicating how they were calculated

Our web collection on statistics for biologists contains articles on many of the points above.

Software and code

Policy information about availability of computer code

Data collection	No software was used for collecting the genomic data.

Data analysis

The following software was used for data analysis:
EdgeR (v.3.6.2)
Pisces (v.0.1.3.1) (https://doi.org/10.1101/2020.12.01.390575)
DEseq2 (v.1.26.0) (https://doi.org/10.1186/s13059-014-0550-8)
FGSEA (v.1.22.0) (https://doi.org/10.1101/060012)
MSigDB gene set annotations (v.7.5.1)
Graphpad Prism (v.9.2, 9.3.1, 9.5.1)
Analyst (v.1.7) software
CytExpert (v.2.4)
Olympus cellSens (v.3.1.1) software
Harmony High-Content Imaging and Analysis software (v.4.9)
Fusion FX7 imager software
Aperio AT2
Microsoft Excel 2016
HALO image analysis platform (v.3.1.1076.437)
Incucyte SX3
Incusyte SX5
Sequence Detection Systems (SDS) (v.2.4.1)
Living Image (v. 4.7.4) (64-bit)

For manuscripts utilizing custom algorithms or software that are central to the research but not yet described in published literature, software must be made available to editors and reviewers. We strongly encourage code deposition in a community repository (e.g. GitHub). See the Nature Portfolio guidelines for submitting code & software for further information.

Data

Policy information about availability of data

All manuscripts must include a data availability statement. This statement should provide the following information, where applicable:

- Accession codes, unique identifiers, or web links for publicly available datasets
- A description of any restrictions on data availability
- For clinical datasets or third party data, please ensure that the statement adheres to our policy

The data supporting the findings of this study are available from the corresponding author upon reasonable request. Publicly available data used in this study include CRISPR-Cas9 loss of viability screens (DepMap Public 20Q4 and DepMap Public 22Q1), CCLE RNA-seq expression (https://doi.org/10.1038/s41586-019-1186-3), and TCGA RNA-seq expression (2019 Q2 release of Omicsoft TCGA B38 gene CPM dataset). RNA-seq data following INPP5A knockdown have been deposited at SRA database with accession code PRJNA860930.

Research involving human participants, their data, or biological material

Policy information about studies with human participants or human data. See also policy information about sex, gender (identity/presentation), and sexual orientation and race, ethnicity and racism.

Reporting on sex and gender	Samples were collected from de-identified patients and stored by the Biobank after obtaining a written informed consent. Sex and gender were not disclosed to researchers and were not considered in study design.
Reporting on race, ethnicity, or other socially relevant groupings	Reporting on race, ethnicity, or other socially relevant groupings was not disclosed to researchers. Samples were chosen based on other selection criteria including oncogenic mutation, tumor cell content, time of collection and availability in the biobank.
Population characteristics	Patients included in this study had melanoma tumors of 2 different subtypes, one with cutaneous melanoma and the other group with uveal melanoma. Patients were mainly diagnosed with distant metastases and who underwent surgical resection between 2019 and 2021 were included. Biopsies from the uveal melanoma group carried mutations in either GNAQ or GNA11 genes and cutaneous melanomas harbor any mutations in BRAF, NRAS or cKIT genes but not in the GNAQ or GNA11 genes. Patients were between ages 60-85 and were not selected based on their current or previous treatment for this study.
Recruitment	This was a retrospective study in which no recruitment underwent specifically for this study. Samples were collected by the Department of Dermatology, University Hospital Zurich, Switzerland. Samples were collected and stored by the Biobank after written informed consent of the patients was obtained (Biobank BASEC-NR_2017-00494).
Ethics oversight	The research presented in this study complies with all relevant ethical regulations and was approved by the local ethics committee (Ethikkommisslon Nordwest und Zentralschweiz (EKNZ) authorization nr. 2021-02243) in accordance with 'good clinical practice' (GCP) guidelines and the Declaration of Helsinki. All biopsies were obtained from patients following a written consent.

Note that full information on the approval of the study protocol must also be provided in the manuscript.

Field-specific reporting

Please select the one below that is the best fit for your research. If you are not sure, read the appropriate sections before making your selection.

☒ Life sciences ☐ Behavioural & social sciences ☐ Ecological, evolutionary & environmental sciences

Life sciences study design

All studies must disclose on these points even when the disclosure is negative.

Sample size	No statistical analysis was performed to predetermine sample size. Sample sizes for individual experiments were determined with standard practices in the respective techniques based on previous experiences and they were chosen to allow biological significance to be detected. Otherwise, pilot experiments were performed to determine assay variability and conditions. The number of independent replicates for each experiment is noted in the corresponding figure legend. Where appropriate, biological replicates (n = 2 or 3) were included, otherwise independent cell lines, sgRNAs or shRNAs were used to confirm our results. For in vivo experiments, no statistical test was used to predetermine sample sizes. Due to intrinsic higher variability, in vivo experiments included at least 4 mice per group. Sample sizes were determined based on previous experience.
Data exclusions	Fig. 6a one mouse was excluded because it did not develop a tumor. Fig. 6d one mouse died due to anesthesia. Few human tumor biopsies were excluded from the analysis according to pre-established quality control (inclusion) criteria that included tumor cell content, biopsy size based on weight (mg), sample handling and collection date.
Replication	All experiments are representative of at two or three biological replicates, otherwise independent cell lines, shRNAs or sgRNAs were used to confirm the results. Detailed information on replicates is available in the figure legends. We were able to replicate all data present in the manuscript. For In vivo experiments, we used enough number of mice to provide statistical power to the differences we observed between control and treated groups.
Randomization	Animals were randomized within their genotype into vehicle or treatment arms prior to initiation of treatment.
Blinding	For all experiments, the experimentalist was not performing data collection and analysis in a blind manner. It is practically not feasible to perform the experiments blindly due to difficulty of planning and execution. Multiple experimentalists were running similar experiments orthogonally either on different cell lines or different assay (e.g. sgRNA or shRNA) to independently confirm results. In addition, for the majority of experiment, different people were collaborating by performing parts of the same experiment to minimize human mistakes and increase integrity, and quantification was mainly done automatically by different machines.

Reporting for specific materials, systems and methods

We require information from authors about some types of materials, experimental systems and methods used in many studies. Here, indicate whether each material, system or method listed is relevant to your study. If you are not sure if a list item applies to your research, read the appropriate section before selecting a response.

Materials & experimental systems

n/a	Involved in the study	
☐	☒	Antibodies
☐	☒	Eukaryotic cell lines
☒	☐	Palaeontology and archaeology
☐	☒	Animals and other organisms
☒	☐	Clinical data
☒	☐	Dual use research of concern
☒	☐	Plants

Methods

n/a	Involved in the study	
☒	☐	ChIP-seq
☐	☒	Flow cytometry
☒	☐	MRI-based neuroimaging

Antibodies

Antibodies used	rabbit anti-GNAQ (1:1000, D5V1B, Cell signaling Technology, 14373)<br>mouse anti-α-Tubulin (1:1000, Sigma-Aldrich, T9026)<br>mouse anti-α-Actinin (1:1000, E7U1O, Cell signaling Technology, 69758)<br>goat anti-ERLIN2 (1:500, Novus Biologicals, NB100-1884)<br>mouse anti-IP3R3 (1:1000, BD Biosciences, 610312)<br>rabbit anti-IP3R1 (1:500, Novus Biologicals, NB120-5908)<br>rabbit anti-cleaved PARP (1:1000, Cell signaling Technology, 9541)<br>rabbit anti-p53 (1:500, Cell signaling Technology, 2527)<br>rabbit anti-p53-phosphoSer15 (1:1000, Cell signaling Technology, 9284)<br>mouse anti-p21 (1:1000, DCS60, Cell signaling Technology, 2946)<br>rabbit anti-HA (1:1000, Abcam, ab9110)<br>mouse anti-Actin (1:20000, C4, Millipore, MAB1501)<br>rabbit anti-BAP1 (1:1000, Cell Signaling Technologies, D7W70, 13271)<br>rabbit anti-pERK (1:1000, Cell Signaling Technologies, 9101)<br>rabbit anti-pRASGRP3 (1:1000, Abcam, 124823)<br>mouse anti-Cas9 (1:300, Cell signaling Technology, 7A9-3A3, 14697)<br>rabbit anti-human Ki76 (1:2000, SP6, Neomarker, RM9106)

Validation

Antibodies were not orthogonally validated in-house. Antibodies with as many trusted citations as possible were used. All antibodies functioned as expected for their respective assays and included positive or negative controls.

Eukaryotic cell lines

Policy information about cell lines and Sex and Gender in Research

Cell line source(s)	Cell lines were obtained from ATCC: A375 (CRL-1619), MeWo (HTB-65), MP41 (CRL-3297), and MP46 (CRL-3298), Sigma: MEL202 (13012457), DSMZ: RHV421 (ACC127), Leiden University Medical Center – M.J.Jager: 92.1 (CVCL_8607), OMM1 (CVCL_6939), OMM2.5 (CVCL_C307), Mel285 (CVCL_C303), and Mel290 (CVCL_C304), or Cancer Cell Line Encyclopedia (CCLE): Colo741 (CVCL_1133), K029ax (CVCL_8784), UACC62 (CVCL_1780)
Authentication	All cell lines were authenticated by SNP profiling and were only used <15 passages to avoid genetic drifting and potential cross contamination.
Mycoplasma contamination	Cell lines were regularly confirmed to be negative for mycoplasma contamination by PCR testing.
Commonly misidentified lines (See ICLAC register)	No commonly misidentified lines were used in this study.

Animals and other research organisms

Policy information about studies involving animals; ARRIVE guidelines recommended for reporting animal research, and Sex and Gender in Research

Laboratory animals	This study used female, 6-8 week old, 20-25 grams, athymic nude mice obtained from The Charles River Laboratories and 6-8 weeks of age NOD.Cg-Prkdcscid Il2rgtm1Wjl/SzJ (NSG) female mice obtained from Taconic Biosciences. The animals were kept under OHC conditions in Allentown mice cages (maximum of 5 animals/cage) with a 12-h dark, 12-h light conditions and they were fed sterilized food and water ad libitum.
Wild animals	This study did not use wild animals.
Reporting on sex	A total of 191 female mice were used in this study. This study only used female mice.
Field-collected samples	This study did not employ filed collected samples.
Ethics oversight	Animal studies were approved by the Cantonal Veterinary Office of Basel Stadt, the local ethics committee Ethikkommisslon Nordwest und Zentralschweiz (EKNZ) and were performed in accordance with the Federal Animal Protection Act and the Federal Protection Order. According to the licence, the maximal permitted tumor size was 1500 mm3, 15 % reduction of body weight or clinical signs of illness. Non of the humane endpoints were exceeded in the study. Source data for all animal experiments are provided.

Note that full information on the approval of the study protocol must also be provided in the manuscript.

Flow Cytometry

Plots

Confirm that:

☒ The axis labels state the marker and fluorochrome used (e.g. CD4-FITC).

☒ The axis scales are clearly visible. Include numbers along axes only for bottom left plot of group (a 'group' is an analysis of identical markers).

☒ All plots are contour plots with outliers or pseudocolor plots.

☒ A numerical value for number of cells or percentage (with statistics) is provided.

Methodology

Sample preparation

Apoptosis assay: Cells expressing doxycycline-inducible control shRNA or two independent shRNA targeting INPP5A (shINPP5A#1 and shINPP5A#2) were treated with either 0 or 100 ng/mL doxycycline for 3 days prior seeding in 96 well plates. Medium with doxycycline was refreshed every 3 days. At the indicated time points, cells were washed with cold PBS, trypsinized, and collected with the supernatant. Apoptosis was determined using the Alexa Fluor 647 annexin V/Dead Cell Apoptosis 34 kit (Invitrogen, V13245 and A23204) according to the manufacturer's protocol. In brief, the samples were incubated with 100 µl apoptosis reagent consisting of 97.5 µl of 1X annexin-binding buffer, 0.5 µl 100 µg/ml propidium iodide, 2 µl of Alexa Fluor 647 annexin V for 15 minutes on ice while protected from light. Stained cells were examined with flow cytometry using the CytoFLEX S analyzer (Beckman Coulter). 3000 events per sample were recorded and gating was set according to single cell distribution. Untreated cells and staurosporine treated cells (17 hours treatment) were used as the negative and positive control for apoptosis, respectively.
Cell cycle analysis: For cell cycle analysis, 92.1 cells were seeded in 6-well plates. 16 hours post seeding, 0 or 100 ng/mL doxycycline was added to the cells. At the indicated time points, cells were pulsed with 10 µM EdU (Sigma-Aldrich, BCK-FC647) for 1 hour before harvesting. The samples were processed according to the manufacturer's protocol. Samples were

incubated with 3 µM 4′,6-diamidino-2-phenylindole (DAPI) for 5 minutes prior to their imaging by flow cytometry using the Cytoflex system (Beckman Coulter).
For FACS-based proliferation assays: Cas9-expressing HEK293A cells were infected with lentiviral particles of the indicated sgRNAs. 3 days post-infection, cells were harvested and 10,000 cells were plated into two 96-well plates (6-8 replicates per condition) and treated with DMSO or 10 nM of FR900359 if applicable. 6 days post-infection, baseline levels of sgRNA-expressing cells were determined by measuring the fraction of mCherry+ or GFP+ cells using a FACS Calibur (BD Biosciences).

Instrument	CytoFLEX S analyzer, Beckman Coulter.
Software	CytExpert (v.2.4).
Cell population abundance	No sorting was performed in this study.
Gating strategy	As describe in method section and the figure legends of Extended Data Fig. 8h, debris and dead cells were excluded according to the forward scatter-area (FSC-A) and side scatter-area (SSC-A) gating. Singlets were identified based on FSC-A and forward scatter-height (FSC- H) profiles. Then they were analyzed for propidium iodide (DNA content) and 5-ethynyl-2 deoxyuridine (EdU) coupled to Alexa Fluor 647 (EdU–647) staining intensities. As EdU is incorporated during active DNA synthesis, EdU+ cells were classified as 'S phase'. EdU– cells were classified as 'G1 phase' or 'G2/M phase' based on their DNA content.

☒ Tick this box to confirm that a figure exemplifying the gating strategy is provided in the Supplementary Information.
