# Supplementary figures and images for: INPP5A phosphatase is a synthetic lethal target in GNAQ and GNA11-mutant melanomas
Source: Nat Cancer. 2024 Jan 17;5(3):481–99. doi: 10.1038/s43018-023-00710-z (PMC10965444; doi:10.1038/s43018-023-00710-z)

Extended Data Fig. 2b

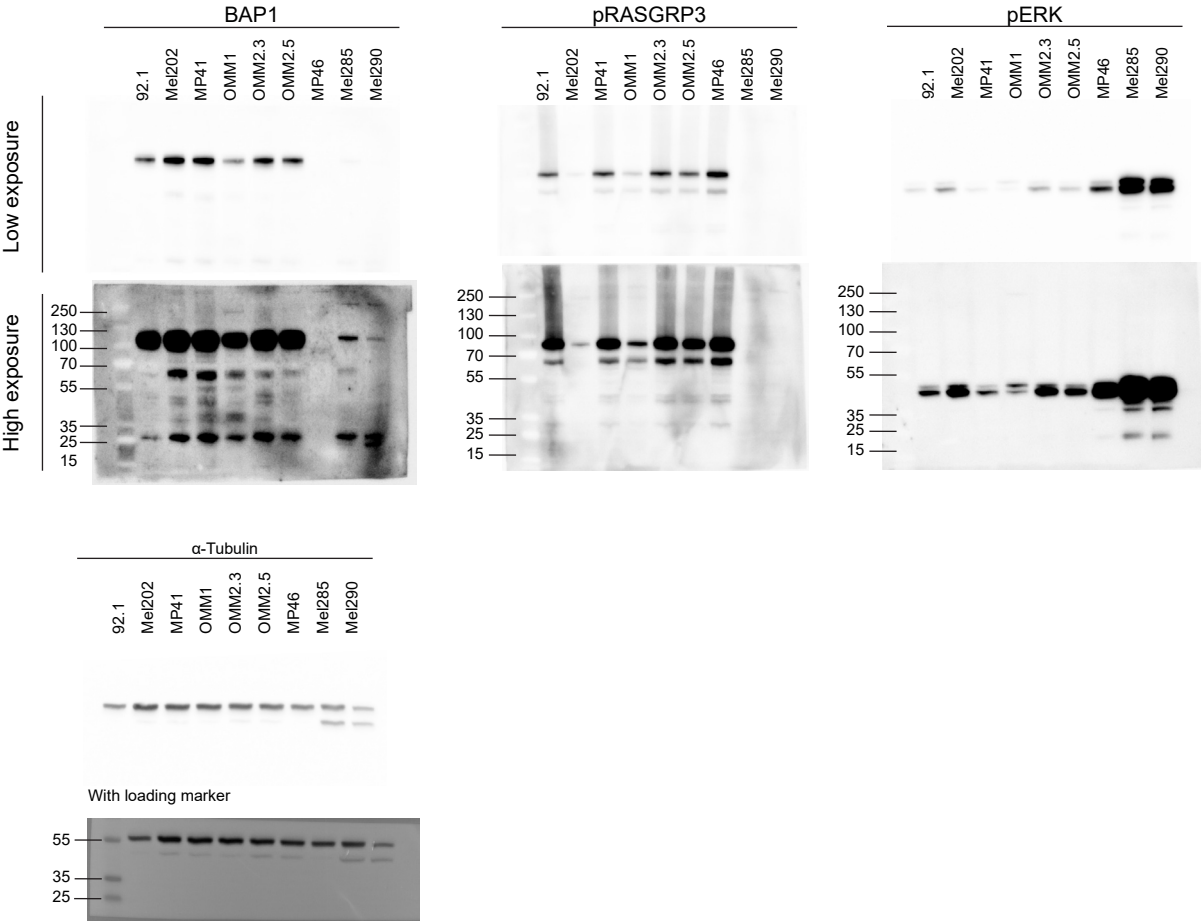

Supplement: Supplementary file 5 — Unprocessed western blots. [file 43018_2023_710_MOESM5_ESM.pdf]

Figure 3f

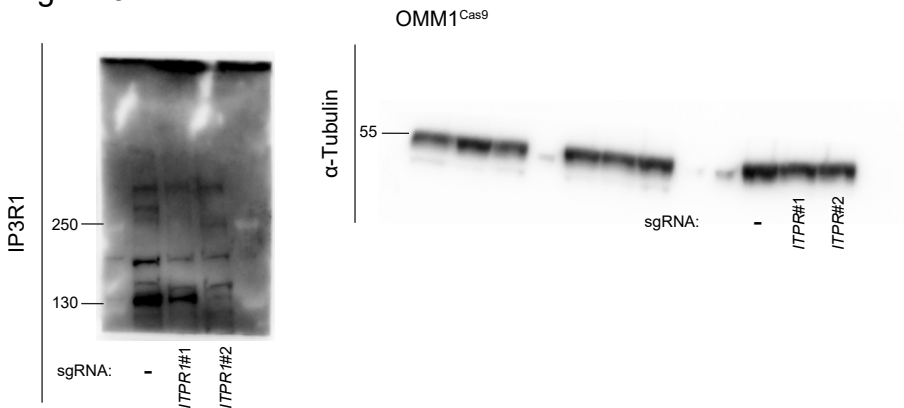

Figure 3g

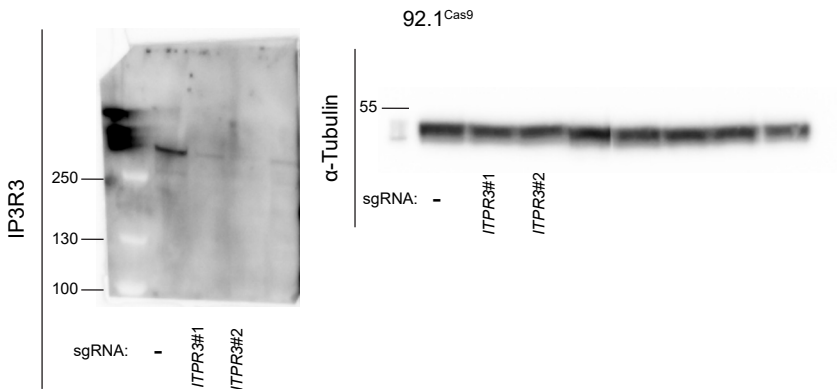

Figure 3k

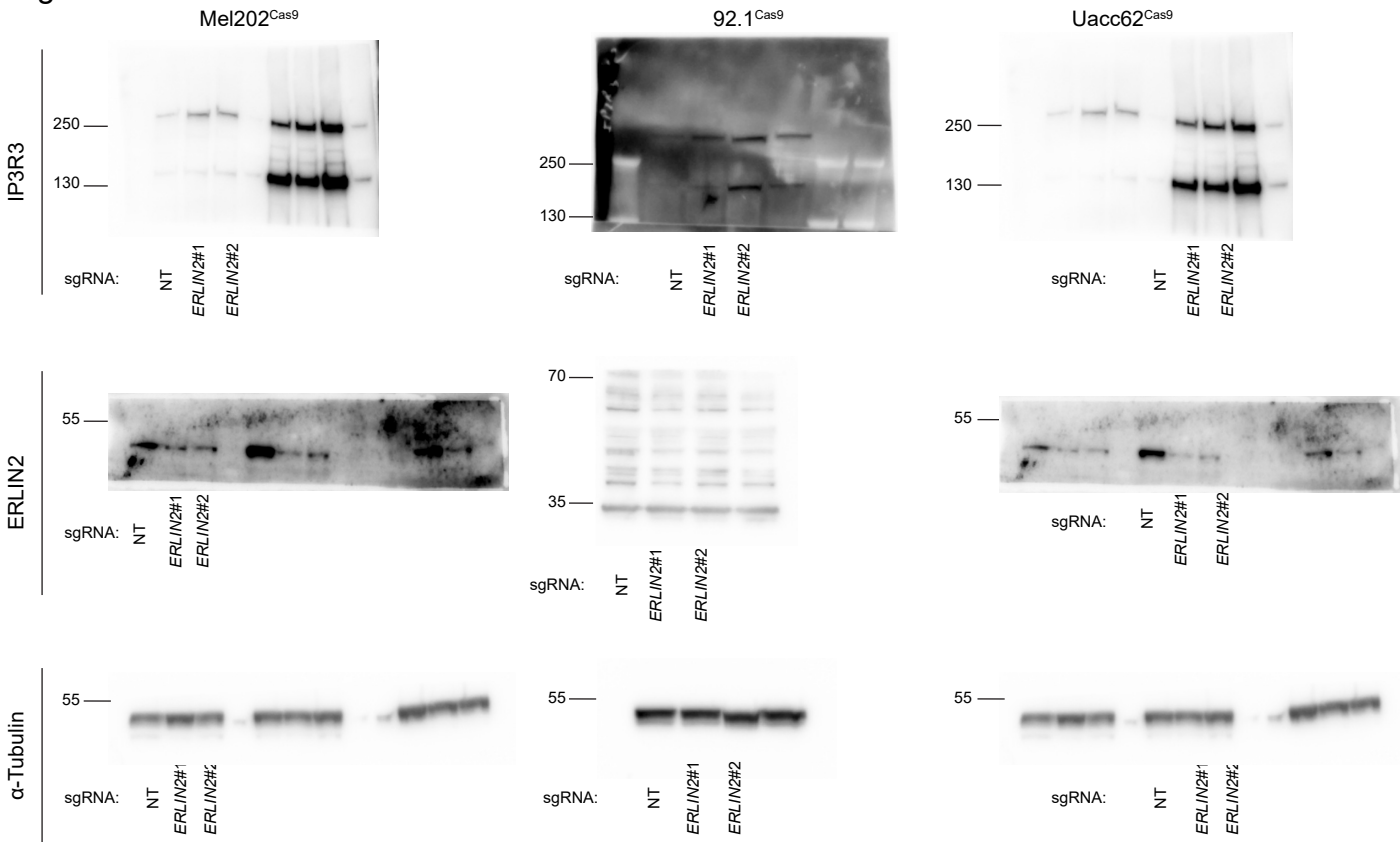

Supplement: Supplementary file 7 — Unprocessed western blots. [file 43018_2023_710_MOESM7_ESM.pdf]

Figure 5d

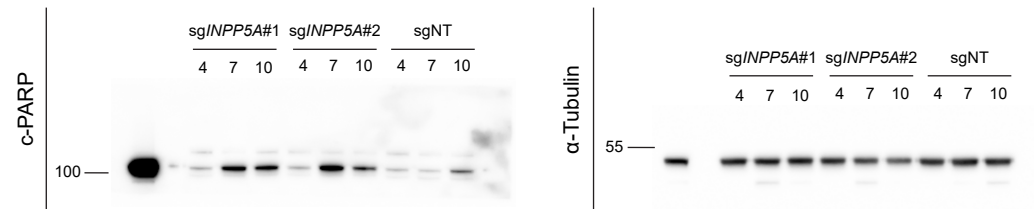

Figure 5f

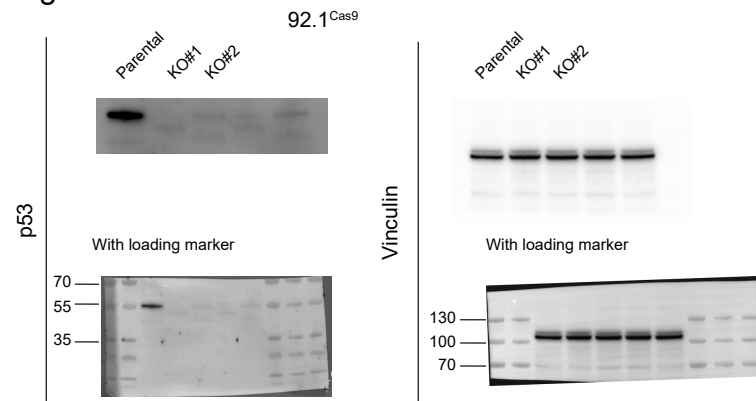

Figure 5g

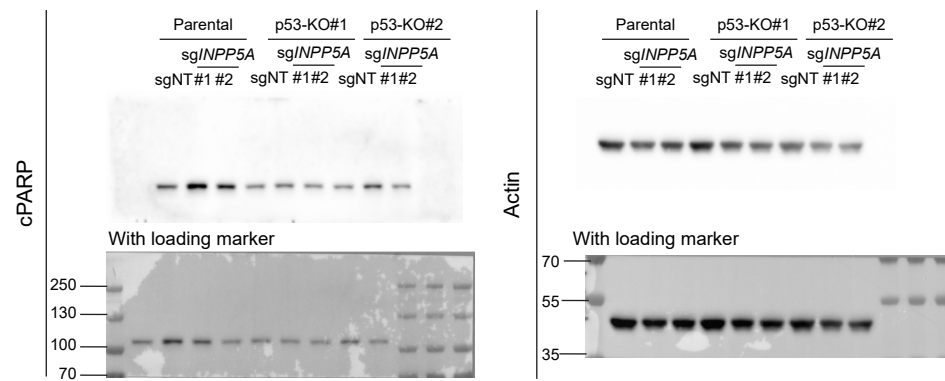

Supplement: Supplementary file 10 — Unprocessed western blots. [file 43018_2023_710_MOESM10_ESM.pdf]

Extended Data Fig. 2b

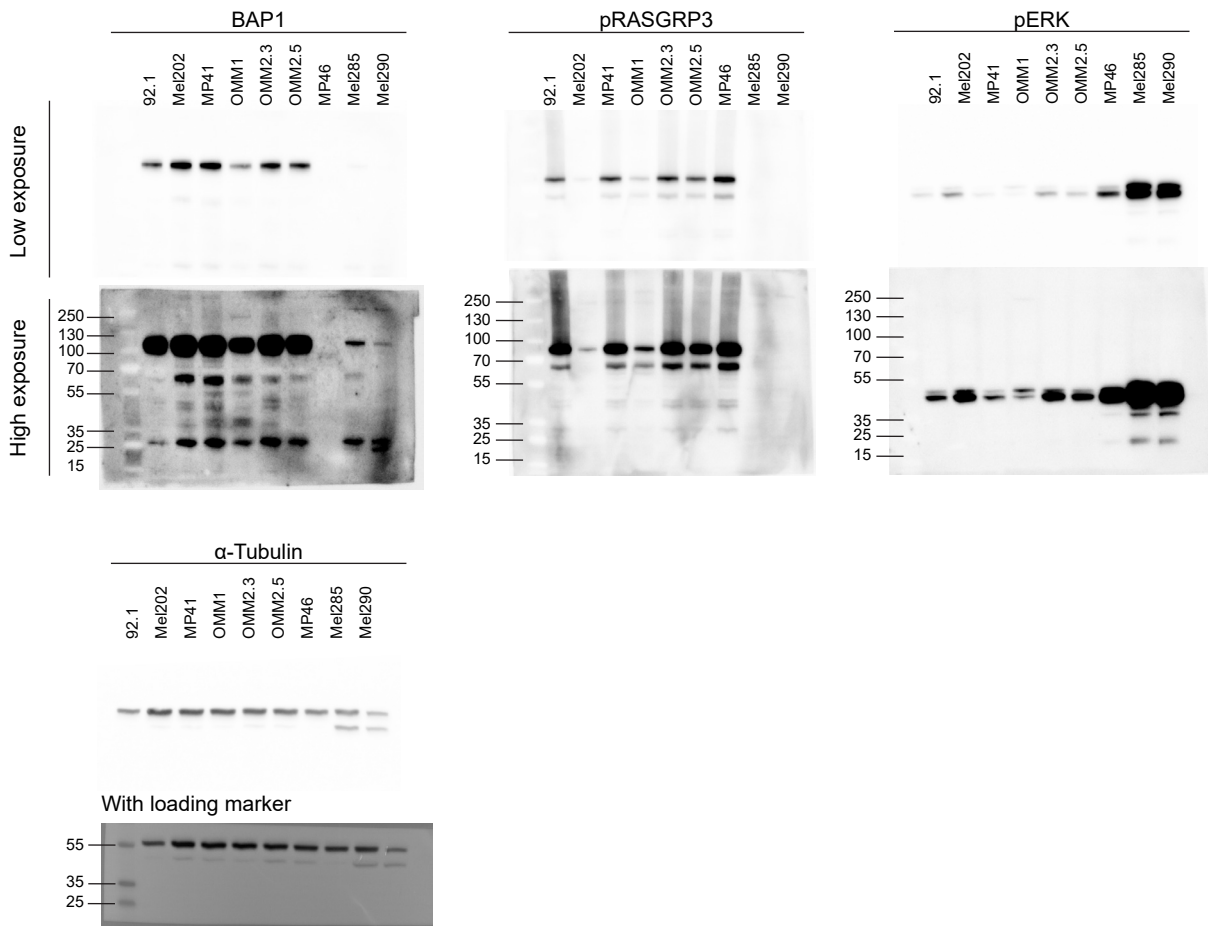

Supplement: Supplementary file 15 — Unprocessed western blots. [file 43018_2023_710_MOESM15_ESM.pdf]

Extended Data Fig. 3h

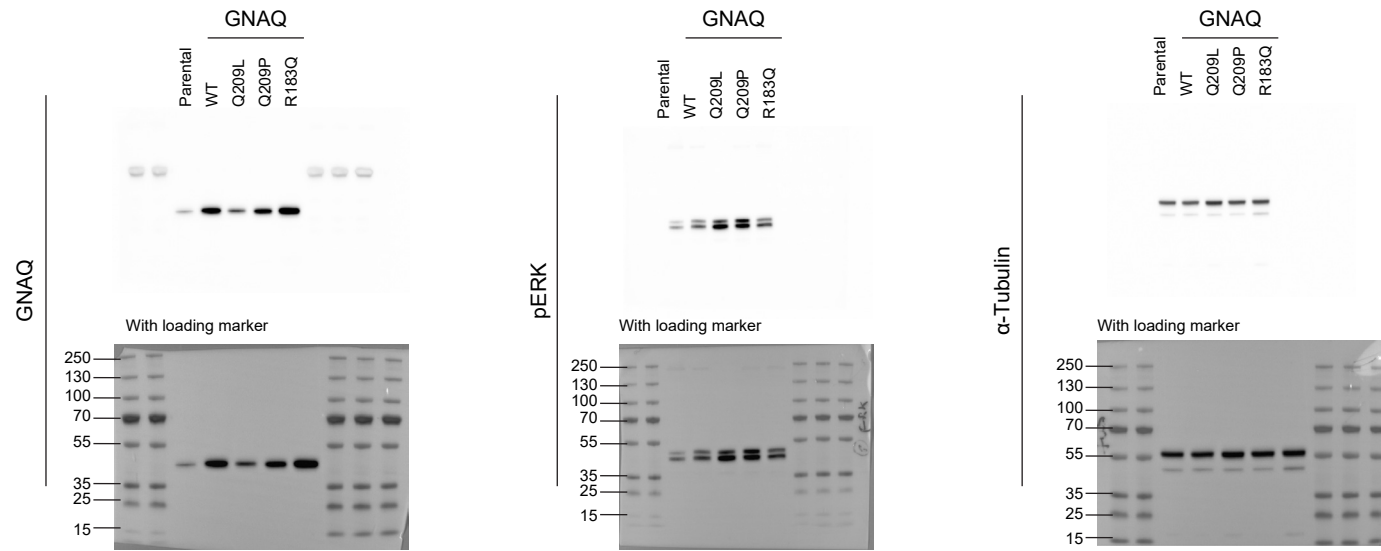

Supplement: Supplementary file 17 — Unprocessed western blots. [file 43018_2023_710_MOESM17_ESM.pdf]

Extended Data Fig. 8d

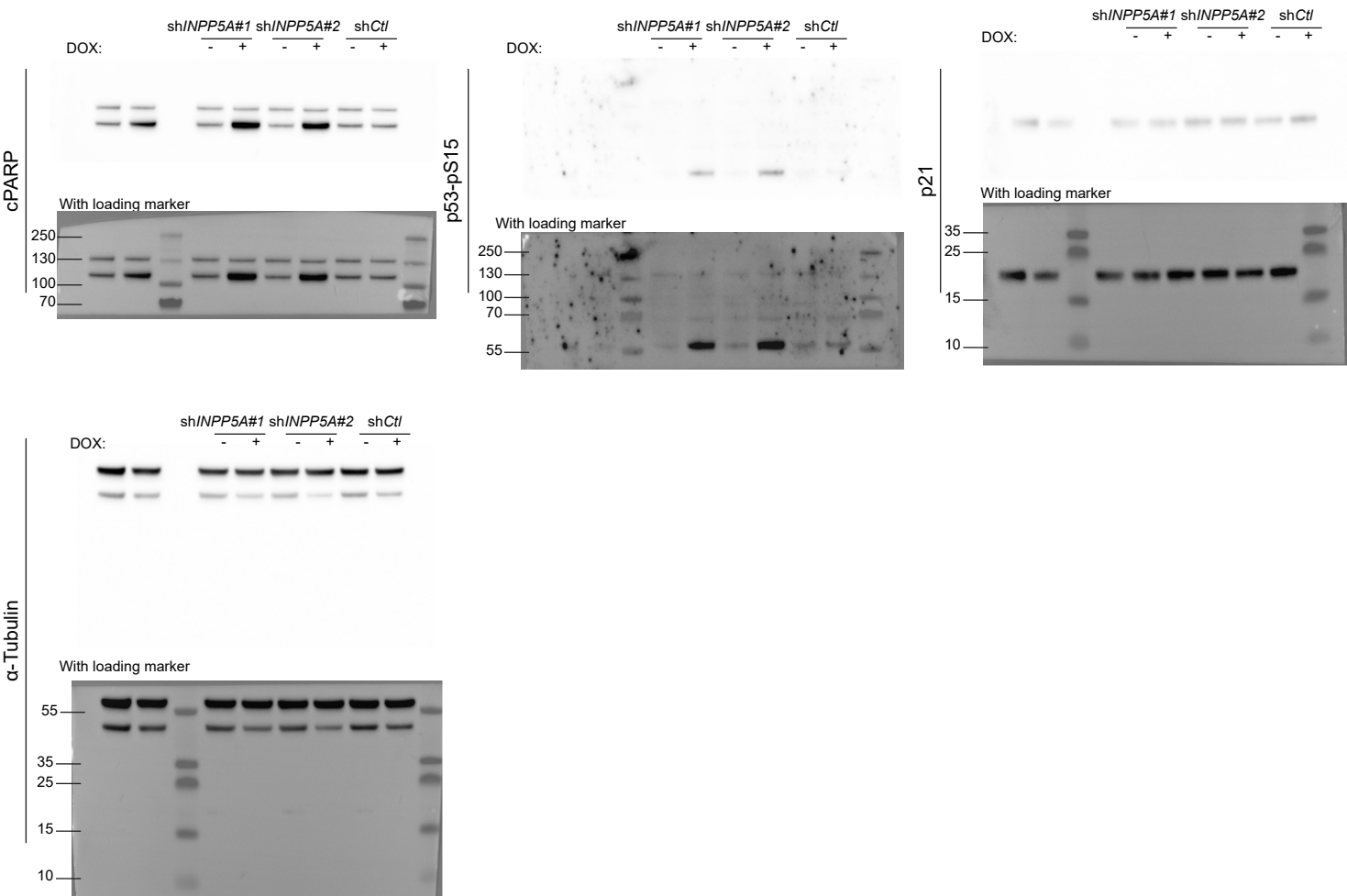

Supplement: Supplementary file 23 — Unprocessed western blots. [file 43018_2023_710_MOESM23_ESM.pdf]
